# Supplementary material for: Evolution of a System to Monitor Infant Neuromotor Development in the Home: Lessons from COVID-19
Source: Healthcare (Basel). 2023 Mar 7;11(6):784. doi: 10.3390/healthcare11060784 (PMC10048217; doi:10.3390/healthcare11060784)
Supplement: Supplementary file 1 [file healthcare-11-00784-s001.zip › Supplement A - AIM Home Parent-Caregiver Manual v5_for Healthcare.pdf]

Assessing Infant Motor Development in the Home: AIM Home

Parent-Caregiver Guide

Table of Contents

Parent-Caregiver Guide 1
Study Overview 3
Background 3
Purpose 3
The Role of Parents/Caregivers in Home Technology Research 3
Study Team 3
General Instructions 4
1. Timing of HUGS Interaction 4
2. How to set up the HUGS system 4
3. How to use HUGS with your baby 11
Data Collection Part 1: Spontaneous Movements 11
Data Collection Part 2: Grasp/attempted grasp in reaction to HUGS toys 12
Parent DATA COLLECTION WORKSHEET 14

0

Study Overview

Background

Sometimes, babies' reaching and grasping (motor) development is delayed but parents/caregivers don't notice a problem. The earlier a baby gets therapy for motor delay, the better the outcome. However, the standard spacing of well-baby visits to the pediatrician makes it less likely that delay will be recognized and therapy started early. To overcome this obstacle, we are developing a low-cost, Hand Use and Grasp Sensor (HUGS) system that parents and caregivers can use at home to help detect motor delay.

Purpose

The purpose of the *AIM Home* study is to enlist the help of parents/caregivers in the design of the HUGS system. It's important for HUGS to capture highly accurate and reliable data but equally essential to make sure HUGS is easy and convent to use in the home.

The Role of Parents/Caregivers in Home Technology Research

Consideration of the patient's priorities in the design of health technology is now best practice in R&D (research and development). In the case of infants, the perspective of the "patient" is especially tied to that of the parent/caregiver. In Aim *Home*, we consider the parents/caregivers who work with us as co-researchers. We are working together to identify what works, what doesn't work, and what can be improved in HUGS system design.

This guide is for parents/caregivers to consult as they work with us to test HUGS in the home. It's a work in progress, and our goal is to continually update it with tips we've learned from *you*, our parent/caregiver co-researchers! If anything is unclear or you encounter any difficulties, please don't hesitate to contact us. A member of the study team is always on call.

Study Team

- Jenny Wang, PhD
- Peter Lum, PhD
- Manon Schladen, PhD
- Harry Kuo, MS

For more information, please contact Principal Investigator: Jing (Jenny) Wang, Ph.D.
Phone: 412-925-4811
EMAIL: wangjin@cua.edu

General Instructions

1. Timing of HUGS Interaction

You know your baby – and your home environment and life flow – best. Some things to consider to help your HUGS session go smoothly are listed below. They may apply to your situation.

- Plan to use HUGS when your baby is calm and alert, not sleepy, fussy, or hungry.
- Plan to use HUGS when you, the parent/caregiver, are not under stress.
- Having another adult or older child to support you could be helpful.
- Check for (and remove) distractions in the room that could interfere with your baby's attention to the HUGS toys: TV/radio/music, ceiling fan, other young children playing. We have seen babies get distracted by these ordinary things in the home environment!
- Make sure that the room in which you choose to do HUGS is well lit, and your baby is not in shadow.
- Make sure that the room in which you choose to do HUGS is not too warm or cold for your baby to be comfortable.

If at any time your infant becomes distressed, please comfort him/her and resume the HUGS test at another time.

2. How to set up the HUGS system

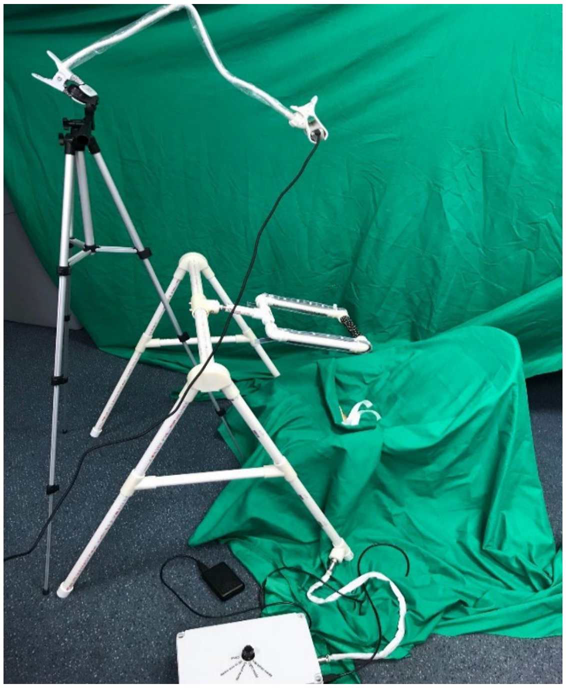

Figure 1. The HUGS system

The HUGS system is composed of three principal parts:

1. the **toys and the frame** from which they are suspended and supporting **electronics**;
2. the **chair** in which your infant will sit during interactions with HUGS (Figure 1); and
3. the **video recording components**, camera frame and laptop-based recording app.

You can follow the instructions below to set up each part of the HUGS system. Detailed descriptions of components of the HUGS system can be found in Table 1.

Frame, Toys, and Electronic Control Box

Step 1:

Connect HUGS Frame with cable (component 1) to the HUGS Electronic Control Box (component 2).

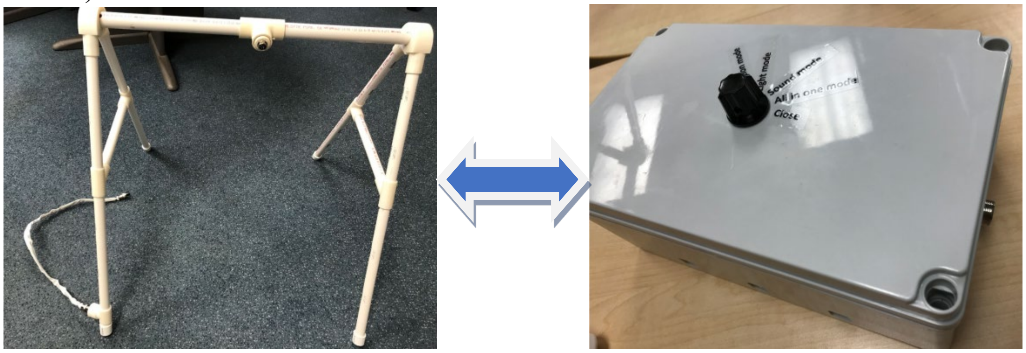

Step 2:

Connect one toy, the Bar Toy (component 3) or the Star Toy (component 4), to the middle of the horizontal bar of the HUGS Frame (component 1) and fasten the connection ring between the toy and the frame.

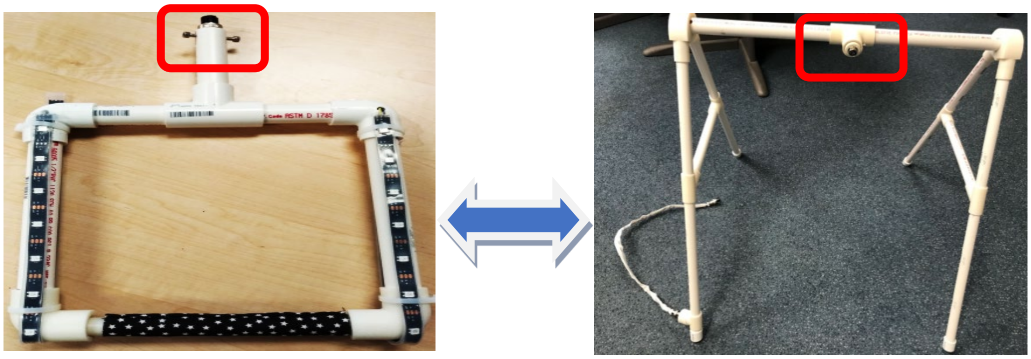

Step 3:

Turn the knob on the HUGS Electronic Control Box (component 2) to choose the feedback mode of the toy. There are three modes: vibration mode, light mode, and sound mode.

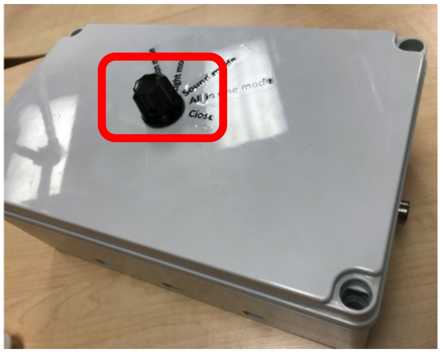

Baby chair

The baby chair will already be at the appropriate angle for your baby based on your work with the research team during your orientation to HUGS. Prepare the chair with stabilizing blankets (as previously determined) and drape it with the green cloth. Put the baby chair on the floor in front of the camera frame.

Video recording components

Step 1:

Turn on the laptop (component 7) and Open *Intel RealSense Viewer* on the desktop of the laptop.

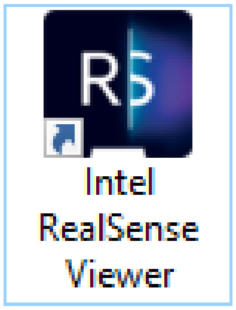

Step 2:

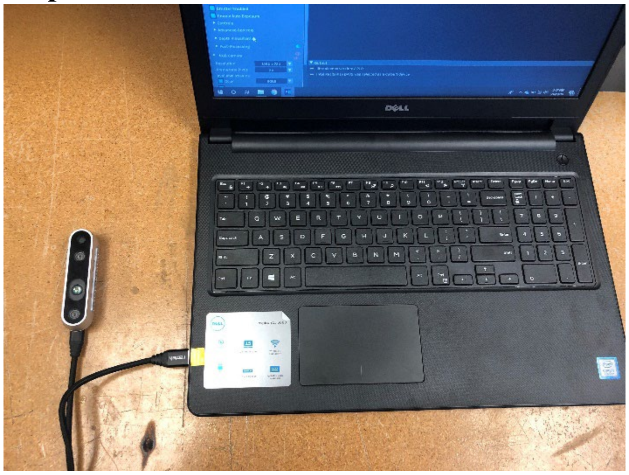

Connect the RealSense camera (component 5) to the laptop (component 7) using the provided cable. Make sure the USB was plugged to the USB port with YELLOW tape on top, on the LEFT side of the laptop.

Step 3:
Turn on the *Stereo Module* and *RBG Camera* tab.

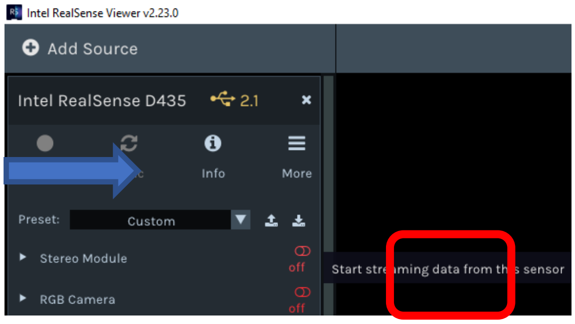

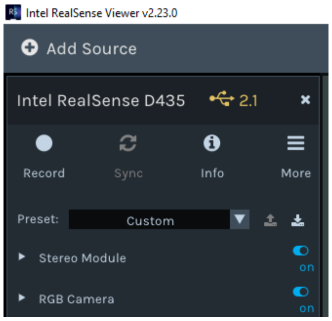

Step 4:
Set up the video frame (component 6) and attach the cameras (components 5 and 6) to it. Adjust the RealSense camera's angle based on the view showed on the laptop. Make sure both the baby chair and the toy are in the middle of the camera view, like the picture below. The image should be as straight and level as possible.

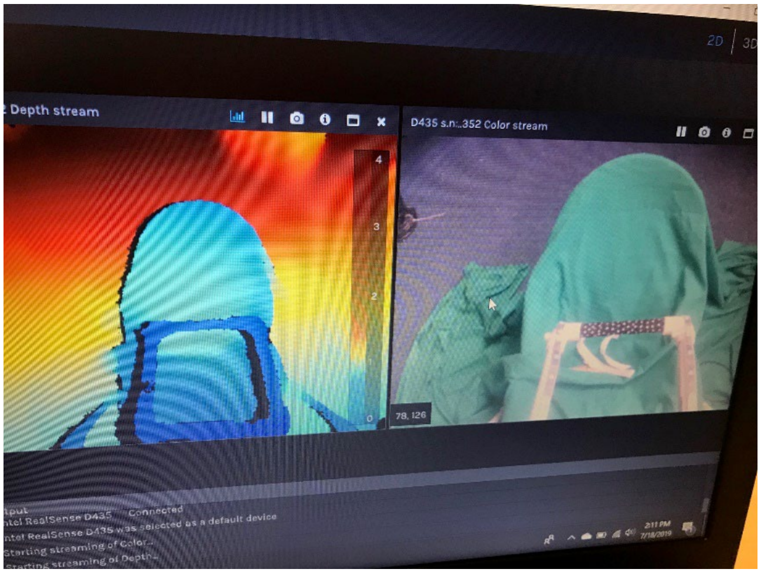

Step 5:
Turn on the ancillary camera It does not need to be connected to the laptop as it uses an SD card to capture your session video and audio. Angle it so it captures the same image as the RealSense camera does.

Now, you are ready to start data collection!

HELP: Do not hesitate to contact the study team if you have any difficulties whatsoever! Our priority is to learn how to make HUGS work better.

Table 1 Components List of the HUGS System

**1. HUGS Frame with cable**<br><br>The interchangeable toys will be suspended from this frame (right) and the angle/height of the toys can be adjusted on this frame for your baby. The cable attached to this frame will be used to connect the electronic control box (Component 2).	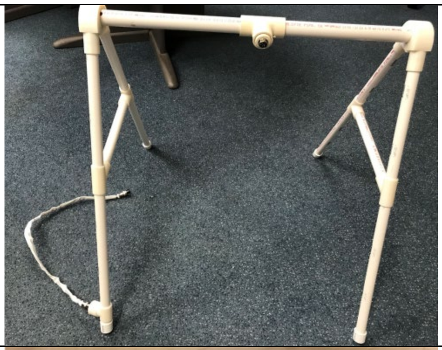
**2. HUGS Electronic Control Box**<br><br>This electronic box will be connected to the HUGS frame above using the cable attached to the frame (Component 1). You can turn the switch on the box to control the type of feedback your baby gets from touching the toys (Components 3 and 4): lights, sound, or vibration. The dial on the control box has five options: vibration mode, light mode, sound mode, and close (turn off). Note: all-in-one more is not used except to test the system.	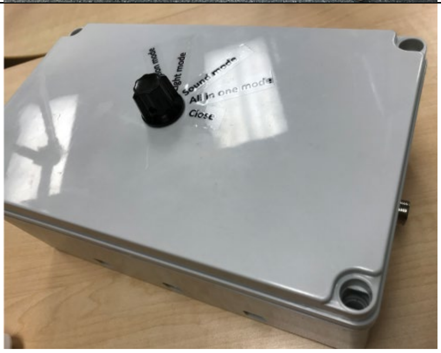
**3. Bar Toy**<br><br>This toy attaches to the frame (Component 1). It is designed to assess your baby's power grasp. It provides feedback to your baby relative to the force of his/her grasp. Feedback is provided through different sensory channels, including touch (vibration), vision (lights), and hearing (sounds). The goal is to study how babies change their hand grasp in response to the different types of sensory feedback HUGS gives them as it detects changes in their grasp force on the toy.	-<br>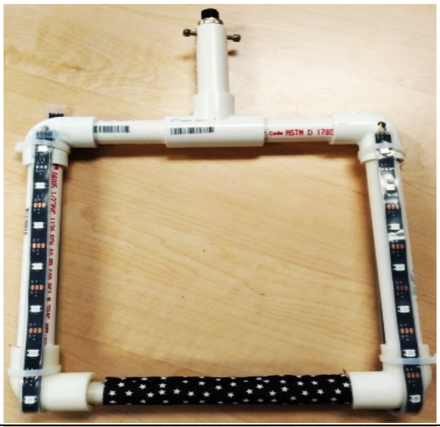
**4. Star Toy**<br><br>This toy, like the Bar Toy (Component 3), also attaches to the frame (Component 1). It is designed to assess your baby's pincer (precision) grasp. Feedback is provided through different sensory channels, including touch (vibration), vision (lights), and hearing (sounds). The goal is to study how babies change their pincer grasp in response to the different types of sensory feedback HUGS gives them as it detects changes in their pincer grasp force on the toy.	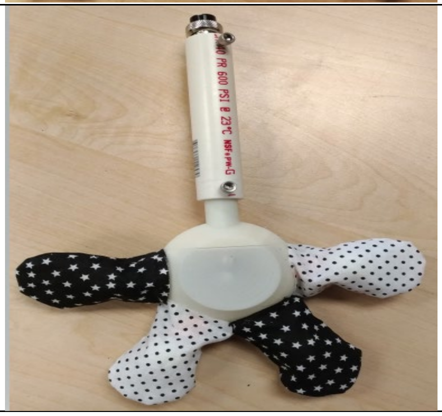

**5. Intel® RealSense™ Depth Camera D435**<br>This camera attaches to the camera frame below (Component 6). It will be used to collect video of your baby's spontaneous movements and interactions with the HUGS toys (Component 3 and 4).	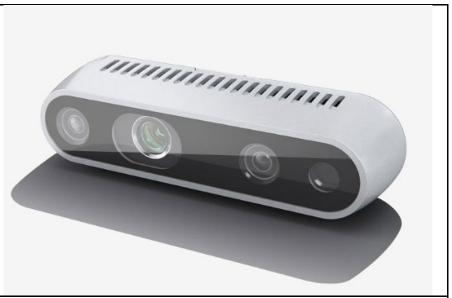
**6. Ancillary Camera**	
**7. Camera Frame**<br><br>This frame will be placed behind the frame that presents the HUGS toys (Component 1) to provide a stable stand for the cameras (Components 5 and 6) for video recording.	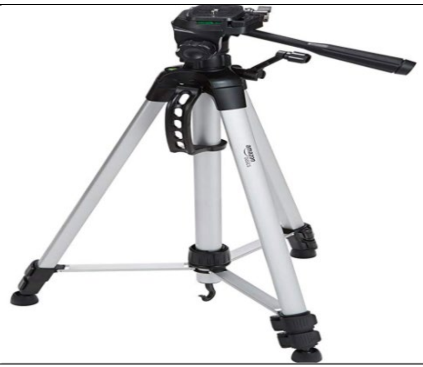
**8. Laptop for video recording**<br><br>This Laptop connects to the RealSense camera (Component 5) using the cable attached. It can be placed on the floor next to the tripod (Component 6). You will use the Intel RealSense Viewer app on the desktop of this laptop to start and to stop video recording as well as to store the video of your baby.	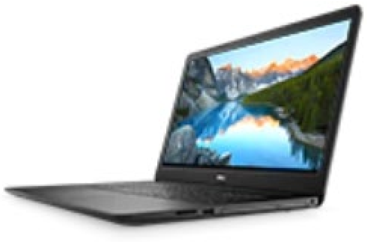
**9. Intel RealSense Viewer**<br><br>You will use the Intel RealSense Viewer app on the desktop of this laptop to start and to stop video recording as well as to store the video of your baby.	
**10. Baby Chair**<br><br>This chair is placed in front of the HUGS frame with a toy attached. You baby sits in this chair to interact with the HUGS system. The chair does not restrict your baby's hands, but keeps him/her centered under HUGS system. The height/incline of the chair can be adjusted to make is easy for your baby to reach the toys. If your baby is very young, you will place rolled up receiving blankets by babu's sides to keep him/her from slumping or slipping sideways in the chair.	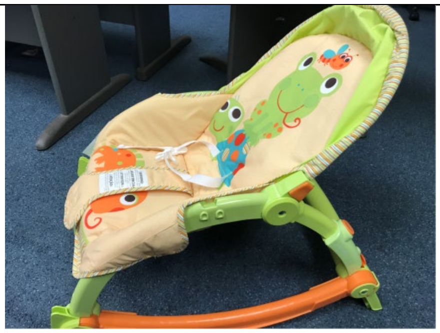

**11. Background Screen**<br><br>This green screen cloth is used as a background for video shooting sessions to make it easier to analyze your baby's video. The green screen cloth goes inside the baby seat, over the stabilization padding (receiving blankets), before you put your baby into the seat. See figure 1.	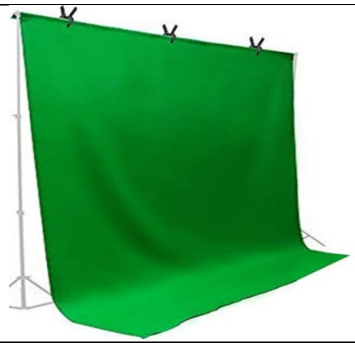
**12. Timer**<br><br>This timer is used to track the time for each data collection session. It is affixed to the camera frame so it is always easy to find.	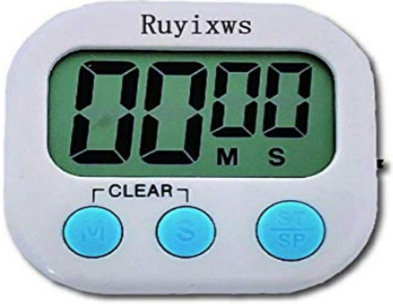

3. How to use HUGS with your baby

You will use HUGS with your baby **FIVE** times during each home trial you choose to participate in. Those five interactions can take place over several days or on a single day. The purpose of the **first interaction** is to capture your baby's spontaneous movements, how s/he moves when not stimulated. The procedure for doing that is described under Data Collection Part 1 below.

During the remaining **four** interactions, you will show your baby how each of the toys works and then give him/her a second opportunity to play with each toy after your introduction. The purpose of providing time between each of these sessions is to make sure, as best you can, that your baby isn't tired or upset by the stimulus of the toys.

Before using the HUGS system, please:

- Dress your baby in the banded, solid white top and green pants provided;
- Place stabilization padding in the chair, as determined was necessary during your orientation session with the research team;
- Place the green cloth over the padding and adjust so your baby will be comfortable;
- Place your baby in the baby chair; and
- Secure the seat belt.

Please use the **WORKSHEET** on the last page to record your data collection activities with your baby. You need to successfully collect the data twice for each toy and once for spontaneous movements. If some issues happen and a data collection session cannot be completed successfully, please record the reason and your comments and find another chance to collect the data with your baby. The instructions for each data collection session are as follows.

If at any time your infant becomes distressed, please comfort him/her and resume the hugs test at another time.

Data Collection Part 1: Spontaneous Movements

The goal is to collect 3 minutes of video of your baby's spontaneous movements, while s/he is sitting (reclined at the angle determined during orientation with the research team) in the HUGS baby chair with no toys or other distraction present. You should not engage (talking, playing, or eye contacting) with your baby during this part of data collection.
Steps to follow:

Step 1:
Start video recording on the laptop.

Step 2:
Set the timer to 3 minutes.

Step 3:

Keep quiet and do not engage (talking, playing, or eye contacting) with your baby during this part of the session.

Step 4:
Stop the video recording on the laptop when the timer rings.

Data Collection Part 2: Grasp/attempted grasp in reaction to HUGS toys

The goal is to measure your baby's grasp and to see which types of feedback – sound, light, or vibration – s/he responds to most strongly. There are two toys to play with: the Bar Toy and the Star Toy. You will do a learning session with your baby for each toy, 2 minutes with each type of stimulus, 12 minutes in total. During this learning session, you will help your baby see what the toys do when touched by placing his/her hands on the toys. We ask you to try to engage your baby as you would typically when playing with him/her. Then, you will repeat the sessions a second time for each toy and each stimulus type: another 12 minutes of recording. This time if your baby doesn't play with the toy for around 30 seconds, please try to encourage him/her as you did in the learning session.

Steps to follow:
Step 1:
Connect the **Bar Toy** on the frame.

Step 2:
Start video recording on the laptop.

Step 3:
Turn the knob on the HUGS electronic box to the **VIBRATION MODE.**

Step 4:
Set the timer to 2 minutes.

Step 5:
Stop the video recording on the laptop when the timer rings.

Step 6:
Repeat the Step 2-5 but turn the knob to choose **LIGHT MODE**.

Step 7:
Repeat the Step 2-5 but turn the knob to choose **SOUND MODE**.

Step 8:
Turn the knob on the on the HUGS electronic box to **CLOSE** and remove the Bar Toy from the frame.

Step 9:
Connect the **Star Toy** on the frame.

Step 10:

Repeat the Step 2-5 to collect data for **VIBRATION MODE**.

Step 11:
Repeat the Step 2-5 but turn the knob to choose **LIGHT MODE**.

Step 12:
Repeat the Step 2-5 but turn the knob to choose **SOUND MODE**.

Step 13:
Turn the knob on the on the HUGS electronic box to **CLOSE** and remove the Star Toy from the frame.

Step 14:
Pull out the cable from the battery and you're all done for the data collection part. Great work and thank you for your effort!

Parent DATA COLLECTION WORKSHEET

	**Video recorded? (Y/N)**	**Date & Time Attempted**	**Successful? (Y/N) If not, any issues?**
**Baby Spontaneous Movement (3 minutes)**			
**Bar Toy Vibration Mode (2 minutes)**			
**Bar Toy Light Mode (2 minutes)**			
**Bar Toy Sound Mode (2 minutes)**			
**Star Toy Vibration Mode (2 minutes)**			
**Star Toy Light Mode (2 minutes)**			
**Star Toy Sound Mode (2 minutes)**			
