# Supplementary material for: Evolution of a System to Monitor Infant Neuromotor Development in the Home: Lessons from COVID-19
Source: Healthcare (Basel). 2023 Mar 7;11(6):784. doi: 10.3390/healthcare11060784 (PMC10048217; doi:10.3390/healthcare11060784)
Supplement: Supplementary file 1 [file healthcare-11-00784-s001.zip › Supplement B - HUGS Project Evolution HUGS-3 Requirements v2 for Healthcare.pdf]

The Departments of Biomedical Engineering and Electrical Engineering and Computer Science
The Catholic University of America

HUGS: Hand Use & Grasp Sensor

Development Phases 1-3

Manon Maitland Schladen 9-15-2022

Background, Rationale for Development 3
Objective 3
Functional Requirements 3
HUGS Evolution 3
HUGS-1 3
Sensory Feedback Modes 3
Toy Configurations Explored 3
Type 1: "Star," "Flower," "Candy" Toy 3
Toy Type 2: "Bar" Toy 4
Affordances of the Bar vs. Candy Toy 5
HUGS-1 Architecture 5
HUGS-1 Operational Components 6
Toys & Suspension (A-Frame) 6
Arduinos (Inside Electronics Box) 6
RealSense RGB + Depth Sensor (3D) Camera 6
Laptop Computer 7
HUGS-2 7
HUGS-2 Operational Components 7
Humans in the Loop 7
GUI 8
Arduinos 8
Bar Toy (+Frame) 8
RealSense RGB + Depth Sensor (3D) Camera 8
OneDrive & Laptop 8
Summary of Experience Using HUGS-1 and HUGS-2: Guidance for the Next System Iteration, HUGS-3 9
1. Footprint 9
HUGS-3 Requirement 1 (Footprint) 9
2. Complexity 10
Connections 10
HUGS-3 Requirement 2 (Complexity) 10
3. Durability 11
HUGS-3 Requirement 3 (Durability) 11
4. File Handling 11
HUGS-3 Requirement 4 (File Handling) 11
5. Force Read Accuracy 12
HUGS-3 Requirement 5 (Force Read Accuracy) 12

6. Ability to Measure Criteria Underlying Clinical Assessment of Hand Use 12
HUGS-3 Requirement 6 (Ability to Measure Criteria Underlying Clinical Assessment of Hand Use) 12
7. Stimulus 13
HUGS-3 Requirement (Stimulus) 13
HUGS-3 14
HUGS-3 Operational View (Mission Level) 16

Background, Rationale for Development

Generally, the younger an infant is when neuromotor delay is detected, the greater the positive impact of therapeutic intervention. It is very common for infants who sustained a perinatal brain injury go unrecognized until well into the second year following birth. Typical, "well baby" visits to the pediatrician do not provide an optimal window for picking up subtle problems in hand use. Infants are seen for only a few minutes every few months and they often present sleepy, hungry, fussy, or apprehensive, "out of phase" with their normal behaviors. Contrast this with the opportunities for detection that exist in the naturalistic environment of the infant's home. Given a means to assess hand use, parents can take multiple measurements, over time, and under conditions where babies demonstrate their usual skills in grasping and manipulating objects.

Objective

To develop, test, and refine an infant Hand Use and Grasp Sensor (HUGS) system for use in the home environment.

Functional Requirements

The Hand Use and Grasp Sensor (HUGS) system has two, over-arching functional requirements:

1. To record the forces generated by an infant's grasp
2. To record infants' hand and arm movements as they grasp objects

HUGS Evolution

There have been two major design phases in HUGS development: HUGS-1 and HUGS-2.

HUGS-1

Sensory Feedback Modes

One of the initial hypotheses was that the type of sensory feedback babies received when they handled a sensorized toy would influence the strength and duration of their grasp. As a result, the protocol called for grasp testing in three modes: visual (flashing colored lights), auditory (music), and haptic (vibration). This hypothesis was not supported by our analysis of data from the HUGS-1 pilot study (2019-2020). However, for consistency we retained the feedback modes and sequential testing of grasp across the modes in HUGS-2 (2021, on-going). We further note that the finding that infants didn't modulate their grasp force or time to demonstrate a preference for auditory, visual, or tactile sensory feedback does not mean that sensory feedback has no effect on how attracted the child is to interacting with a toy.

Toy Configurations Explored

Initially, HUGS had **two** basic types of toys for babies to grasp.

Type 1: "Star," "Flower," "Candy" Toy

One type featured spherical protrusions for infants to grasp: the "star" toy. See https://youtu.be/XPpxIo2iEto

A variation flattened the spheres into "petals" to make a "flower" toy.
See https://youtu.be/1vPqQzwN9pU

Within several testing sessions, the flower toy was discontinued and the star toy lost all but two of its spheres due to an inability on the part of the infant to grasp the points of the star at the six o'clock position. This modification was accompanied by a change in name, making the "star" toy a "candy" toy, as displayed in figure 1a.

Toy Type 2: "Bar" Toy

The other type of toy explored in the HUGS-1 system presented the child with a bar for grasping: the "bar" toy. Variations experimented had bars of different lengths and thicknesses. One separated the visual feedback stimulus (i.e. light) from the grasp-sensing bar itself.
See https://youtu.be/NQftbnDDHaI and lab demo https://youtu.be/I5-MM1lSyFo

The later version of the bar toy incorporated the visual stimulus, created by an LED strip, inside the bar. See figure 1b. The toy bar in action can be observed at this link.
https://www.youtube.com/watch?v=vSJ5r09X5gw&t=135s

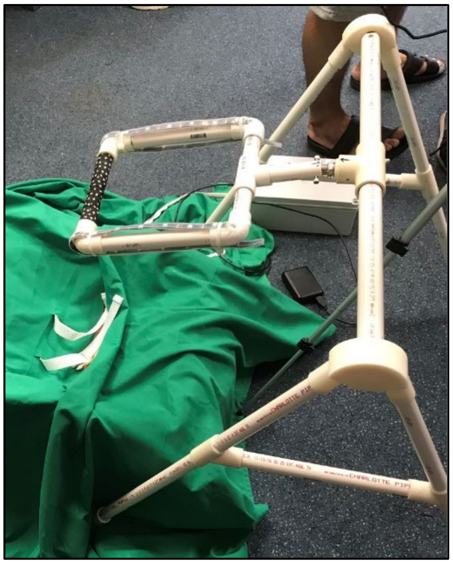

Figure 1. Early rendition of the bar toy with LED light strip on the suspension frame

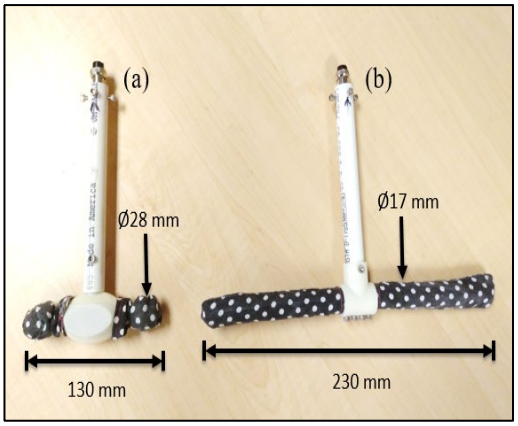

Figure 2. Candy Toy (a) and Bar Toy (b)

Affordances of the Bar vs. Candy Toy

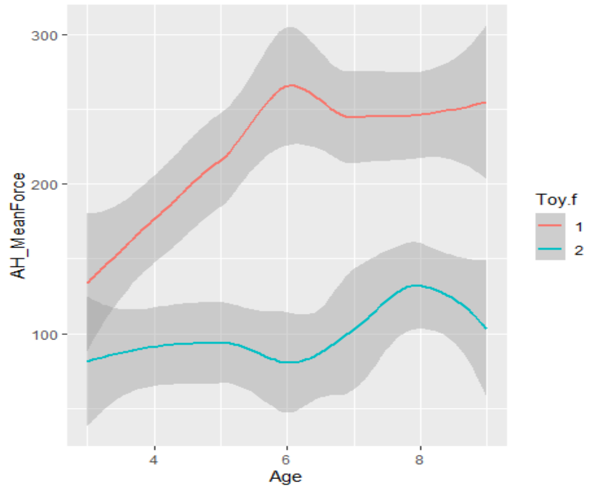

Figure 3. Comparative Mean Grasp Forces, Bar vs. Candy Toy

Both bar and candy toys were tested with typically developing infants during the in-home trial of HUGS-1. The candy toy was discontinued in HUGS-2 as it appeared to diminish infants' performance. See Figure 3. Infants did not or were not able to grasp the candy toy (toy 2) as forcefully as they did the bar toy (toy 1). Note that the "bump" in grasp force occurs at six months as measured by the bar toy and at eight months as measured by the candy toy Infants' weaker performance on the candy versus bar toy was mirrored in recorded duration and frequency of grasp as well.

HUGS-1 Architecture

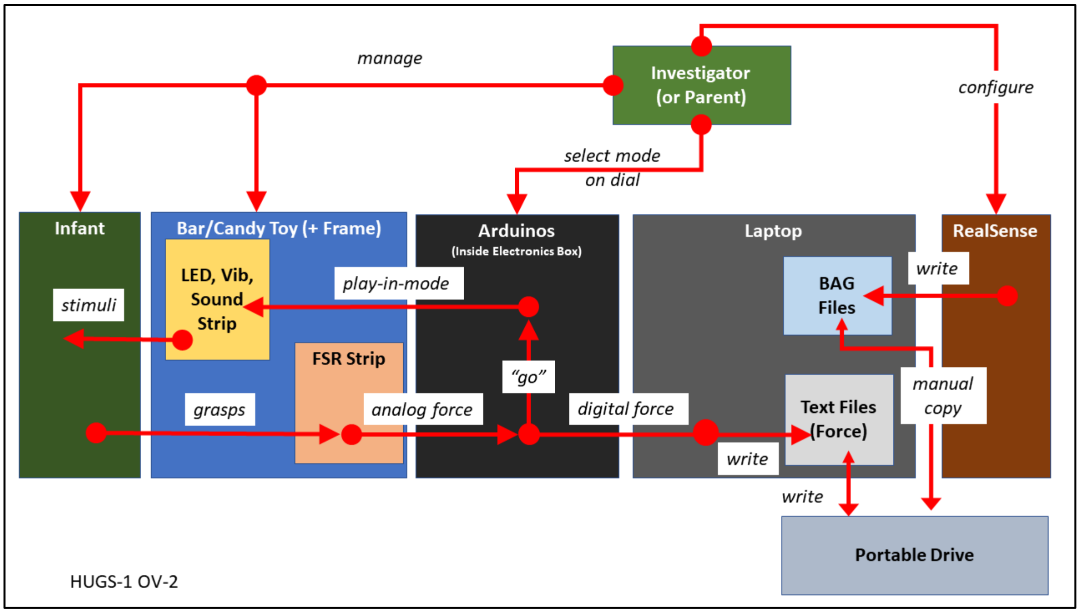

Figure 4. HUGS-1 Operational Resource Flow

The initial HUGS-1 system is shown in Figure 4. This build was used for the first three infants piloting the system. Operational components are described below with notes on the changes that were instituted to the final HUGS-1 configuration as a result of the first three in-home trials.

HUGS-1 Operational Components

Toys & Suspension (A-Frame)

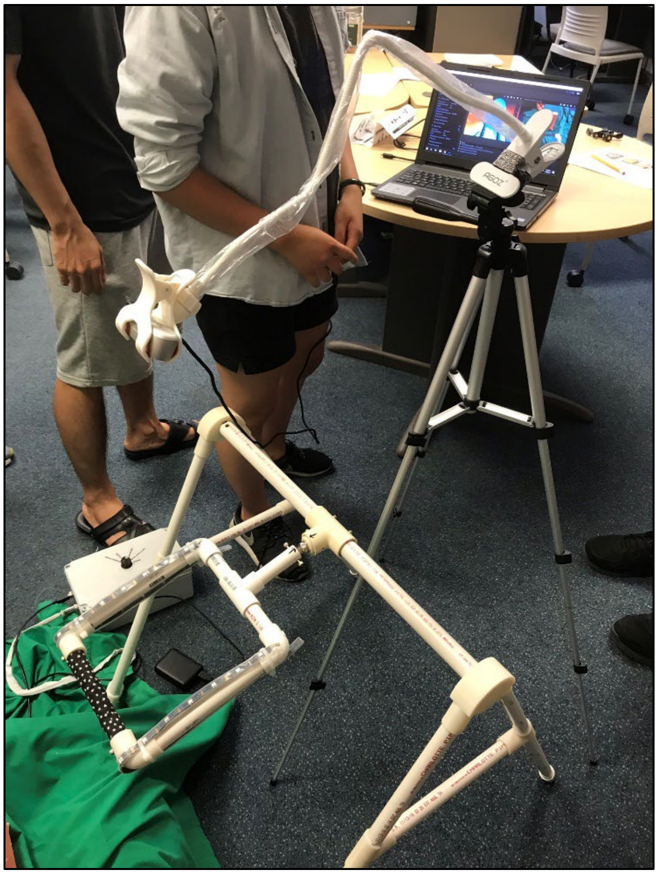
Figure 5. Early HUGS-1 Instance, July 18, 2019

- **Bar Toy.** Figure 4 shows the initial, square-bracket version of the bar toy. Its distinguishing feature is that visual feedback (LEDs) was provided on the descending arms of the square bracket supporting the grasp bar. This design gave way to the bar toy as shown in Figure 1b. The reason for the change was that infants were attracted to the LEDs and tried to grasp them as opposed to grasping the bar (that both actuated the lights as well as recorded their grasp forces). This video shows pilot infant 2 interacting with the early bar toy. https://youtu.be/NQftbnDDHaI

- **Candy Toy.** The candy toy (Figure 1a) was preceded by variants called, respectively, the "star" and "flower" toys. These variants provided multiple protrusions, configured like spokes around the hub of a wheel, for the baby to grasp. Visual feedback (light) when they grasped the "spokes" was provided by lighting up of the "hub."
  - **Star Toy.** This video https://youtu.be/XPpxIo2iEto shows pilot Infant 2 interacting with the star toy.
- **Toy Suspension Frame.** The A-frame supporting the toy bar was fixed, i.e. not able to be collapsed. In HUGS-2, the frame design was changed to allow it to be folded flat for storage when not in use.

Arduinos (Inside Electronics Box)

- Feedback modes were controlled by a dial on the electronics box. The parent/researcher set a standalone, commercial digital timer to determine when to switch stimulus modes. In HUGS-2, an interface to a visual basic program on the laptop provided a GUI (graphical user interface) for parents/researchers to use to select and time the feedback mode the infant would be offered.
- Force data were transmitted from the toy bar to the electronics box Arduinos via a cable connected to wiring that ran through the interior of the A-Frame to the toy bar connector.
- Force data (.txt files) were manually transferred by researchers from the electronics box to an external drive.

RealSense RGB + Depth Sensor (3D) Camera

- The RealSense camera was extended from a single tripod supported by a "claw" at the end of a flexible arm to capture video of the infant. This arrangement proved unstable and a configuration where two camera stands supported a pole attached horizontally between them. The RealSense

camera was then secured to the pole using a clip capable of rotating around the pole to adjust the direction of video capture to frame the infant.

Laptop Computer

- The camera was directly connected to the laptop by USB. It is controlled by an app on the laptop and writes its 3D .BAG files to a folder on the laptop.
- In-lab demonstration of HUGS under the configuration described in the previous paragraph. https://youtu.be/I5-MM1lSyFo

HUGS-2

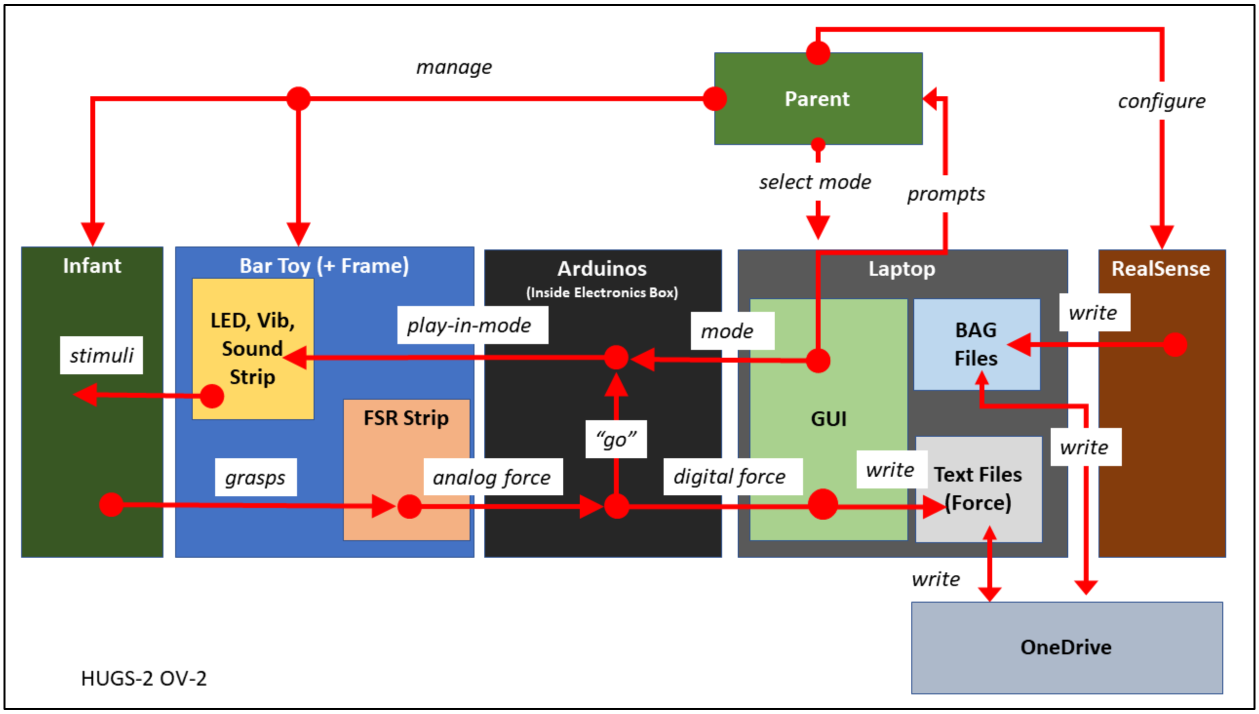

Figure 6. HUGS-2 Operational Resource Flow

Figure 6 shows the operational interfaces among the major components and subcomponents of HUGS-2. Since HUGS-2 was put in the field to capture data from at-risk infants while the COVID-19 lockdown was strictly in force, researchers did not go into the home to support parents in their use of the system. Rather, HUGS-2 was dropped off and researchers provided online, multimedia training and synchronous support over Zoom. The website linked below provided detailed, step-by-step instructions to families to support them in using HUGS to collect their babies' grasp force and spontaneous movement data. https://sites.google.com/cua.edu/hugs/home

HUGS-2 Operational Components

The sections below describe the operation of HUGS-2 as diagramed in Figure 6. Major design modifications from HUGS-1 are noted.

Humans in the Loop

The parent (dark green block, figure 6, top) positioned the infant in the view of the RealSense camera (brown block) and verified correct framing of the infant via the RealSense Viewer app installed

on the laptop computer (gray block). Instead of turning a dial on the electronics box (as in HUGS-1) to select the stimulus (light, sound, vibration) to encourage the infant to grasp the toy bar (blue block), the parent used a custom app (GUI, light green block inside dark gray laptop block).

The infant (dark green block, figure 6 left), in turn, grasped and manipulated the toy bar as desired during the sequential presentation of the three feedback modes as specified from within the GUI. The infant's grasp force was detected by a force sensing resistor (FSR, light orange block) inside the Bar Toy (blue block).

GUI

The GUI (light green block inside gray Laptop block) performed the following operations:

- Prompted the parent to select a feedback mode, specified at random, to engage the infant in manipulating the toy bar;
- Signaled the Arduinos (black block) to set a feedback mode to present the infant in response to grasping the Bar Toy (blue block);
- Received measures of the infant's grasp force from the Arduino array (black block);
- Logged the received grasp force data as text files (light gray block) to the OneDrive partition on the laptop computer (dark gray block).

Arduinos

An Arduino Uno array (black block), housed in an electronics box as in HUGS-1, managed transmissions to and from the Bar Toy under control of the GUI. This design replaced the dial for feedback mode selection under HUGS-1. It also provided the first leg of a process for automated force file transfer to the Cloud (OneDrive, silver block).

Bar Toy (+Frame)

The feedback mode selected by the parent using the GUI was transmitted to the Bar Toy by the Arduinos through wires running through the A-Frame. This configuration persisted from HUGS-1. As previously noted, only the Bar Toy was used with infants testing HUGS-2. LEDs to provide visual feedback on grasp were housed within the Bar Toy itself and the sound and vibration components variously housed within the A-Frame (yellow block inside blue Bar Toy + Frame block).

RealSense RGB + Depth Sensor (3D) Camera

As previously described, the RealSense camera was adjustably mounted on a horizontal pole suspended between two, commercial camera stands. It captured 3D video of the infant and wrote a file in .BAG format for each mode (light, sound, vibration) to the OneDrive partition on the laptop.

OneDrive & Laptop

The Microsoft OneDrive cloud data management application was installed on the HUGS-2 laptop. OneDrive was automatically configured to upload files (.BAG video and .txt force) to the cloud whenever a WiFi connection was available. The laptop was configured to automatically connect to the infant's family WiFi and upload all HUGS-2 data, force and video.

Summary of Experience Using HUGS-1 and HUGS-2: Guidance for the Next System Iteration, HUGS-3

1. Footprint

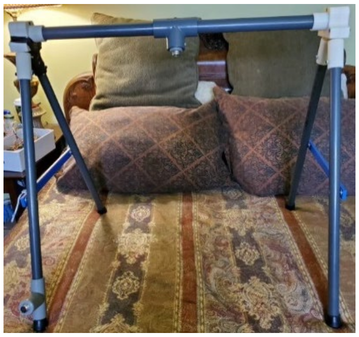

Figure 7. A-Frame Extended

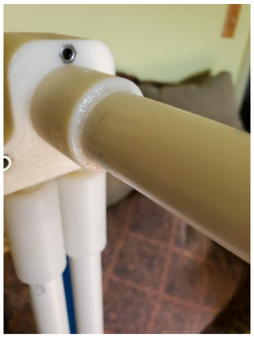

Figure 8. A-Frame Folded

Making the A-frame collapsible did not have the desired effect as parents were reluctant to take the system down once it was assembled. The size of the system became a clear deficit during HUGS-2, when it was left in the home for the duration of testing: up to nine months. During HUGS-1 testing, researchers transported the system to and from the infant's home between sessions. Since families were reluctant to "put HUGS-2 away," it ended up in the path of household activities and breakage was a common occurrence.

HUGS-3 Requirement 1 (Footprint)

1.1. HUGS-3 will be easy for families to put away when not in use.
1.2. HUGS-3 will have a small footprint.

2. Complexity

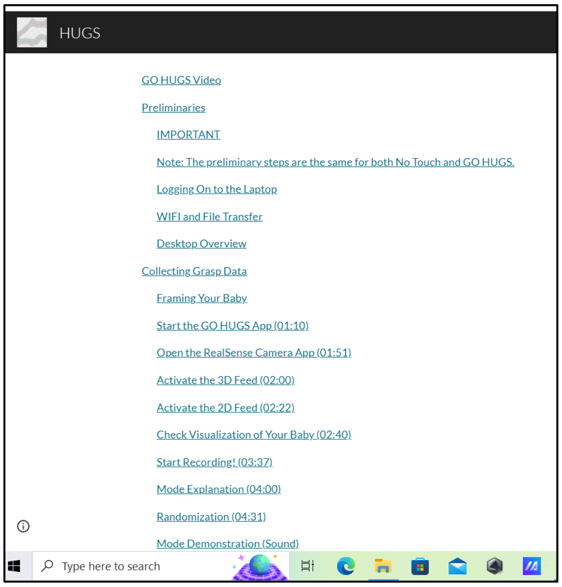

Figure 9. Screenshot of Website Instructions for Capturing Grasp Data. https://sites.google.com/cua.edu/hugs/data-collection/go-hugs

The introduction of a GUI to coordinate data capture added automation to the process but did not necessarily reduce its complexity. The screenshot at the left (figure 9) provides a gestalt of actions required of the parent after automation. They are not significantly less than those required for HUGS-1, though automated processes (i.e. clicking on a button) replace manual ones (i.e. turning a dial). The greatest simplification came in the form of automated data upload to the cloud through leverage of the Microsoft OneDrive application.

The multiple hard connections that had to be made to get HUGS-2 operational were daunting to families precipitating a desire, a previously noted, to simply leave HUGS set up and (in theory) read to use across the multiple months it was in the home.

Connections

- Camera to laptop
- Camera to suspension bar
- Electronics box to A-Frame
- Electronics box to laptop
- Bar toy to A-Frame (note – changed to a hard connection due to frequent failures)

HUGS-3 Requirement 2 (Complexity)

2.1. HUGS-3 will minimize complexity in setup.
2.2. HUGS-3 will minimize complexity in operation.

3. Durability

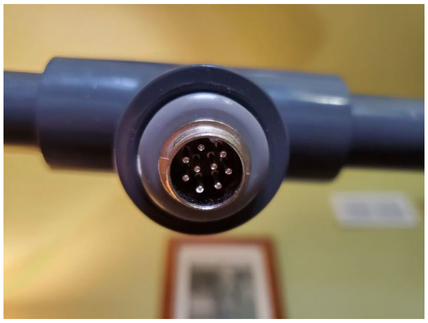

Figure 10. Nine-pin Connector, Horizontal Member of A-Frame (from below)

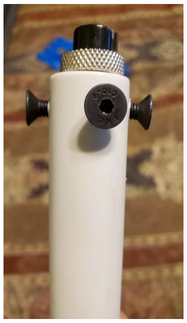

Figure 11. Bar Toy Flange, Female, Nine-pin Connector (lateral view)

Breakage of the frame, and of the Bar Toy itself, was a frequent problem. Most issues of fragility were a consequence of 3D printed components, particularly the pivot brackets that held the legs of the A-Frame and the T-junction of the Bar Toy (figure 8).

The wires, with various nine-pin connectors, that ran from the electronics box, through the A-Frame, and down into the Bar Toy were another common point of breakage, with the most common point of failure being within the Bar Toy or at the final connector (figures 9 and 10). Relatively early in home testing, the decision was made to make the Bar Toy to A-Frame interface a hard connection to reduce breakage.

HUGS-3 Requirement 3 (Durability)

3.1. HUGS-3 will resist being pulled apart by the infant.
3.2. HUGS-3 will be hardened against being dropped or thrown by the infant.

4. File Handling

Transfer of force data from FSR to Arduino to GUI to Laptop storage to cloud storage was robust. Each feedback mode data capture took the form of numerical strings saved in .txt format. Hence file sizes were minimal.

The converse was the case for 3D video data, with .BAG files averaging five GB each. In cases where the parent forgot to cycle the RealSense between modes, capture would be aggregated resulting in .BAG files greater than 10 GB. It was not uncommon for the transfer of files for a HUGS-2 session to take several hours over the family's WiFi with a corresponding several hours' worth of opportunity for transmission errors to occur.

A further problem, related to transmission errors, was running out of space to store the .BAG files on the HUGS-2 laptop. This problem was addressed by moving files for a session once they uploaded to a different folder on OneDrive, one that was not selected to replicate on the laptop in the home. If files could not be uploaded, however, the "cleaning" solution would not be useful in conserving space on the HUGS-2 laptop.

HUGS-3 Requirement 4 (File Handling)

4.1. HUGS-3 will accommodate large file uploads with no data loss.
4.2. HUGS-3 will have ample local storage to hedge problems with file upload.

5. Force Read Accuracy

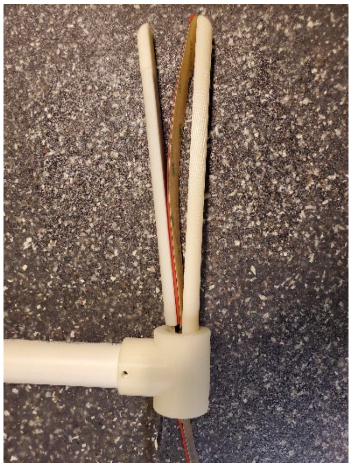

Figure 12. FSR Sandwiched Inside of Bar Toy

The FSR used to detect infant grasp force is a flat strip. It was sandwiched inside two, 3D printed half-round pieces to form a cylindrical bar for the infant to manipulate (figure 11). Because of the FSR's orientation in a single plane, grasp forces coming orthogonal to the FSR would register differently that forces applied laterally or obliquely. It was necessary to observe the infant's grasp from video to compare grasp forces delivered at different angles.

HUGS-3 Requirement 5 (Force Read Accuracy)

5.1. HUGS-3 will measure force uniformly regardless of the position of the infant's hand during grasp.

6. Ability to Measure Criteria Underlying Clinical Assessment of Hand Use

In the time since HUGS development and testing has been on-going, an assessment of infant hand use, the HAI or Hand Assessment in Infants, has been validated. The HAI is well-disseminated in Europe and Australia, and more and more therapists in the United States are beginning to adopt its measures. Administering the HAI requires assembling a "toy kit," a selection of toys that can be presented to an infant in specific ways to try to elicit maneuvers that make up the 17 items on the assessment. Notably, the HAI presumes that infants will be able to freely manipulate and maneuver the toys they grasp. The toys are untethered, in contrast to the HUGS concept where toys are suspended from a rigid bar. Preliminary analysis of grasp data from HUGS-2 revealed very little movement in the vertical since infants' hands were preoccupied with the Bar Toy, which they explored by moving their hands across its surface in the horizontal.

Pose estimation applied to HUGS-2 video demonstrated significantly better recognition of infant hand points of interest (anatomical landmarks) when the video was taken from behind (looking over the baby's shoulder) than when taken from the front. In the view from the front, the Bar Toy itself often occludes the baby's hand landmarks.

HUGS-3 Requirement 6 (Ability to Measure Criteria Underlying Clinical Assessment of Hand Use)

6.1. HUGS-3 will allow free movement of the infant's hand and arm, in any direction, during grasp.
6.2. HUGS-3 will capture infant grasp activity from the front.

7. Stimulus

As previously noted, analysis of data from HUGS-1 did not find any difference in grasp force or duration for the different feedback modes: visual, auditory, and tactile. The three modes were retained in HUGS-2 since no difference among stimuli does not mean that stimulus itself is not useful in attracting a baby's interest and exploration. Notably, the HAI (Hand Assessment for Infants) manual recommends toys for the assessment toy/tool kit that "make a noise." Varied textures are also recommended. There is no recommendation for using light as an attractor, however.

HUGS-3 Requirement (Stimulus)

7.1. HUGS-3 will provide auditory feedback to the infant during grasp.
7.2. HUGS-3 will provide tactile feedback to the infant during grasp.

HUGS-3

HUGS-3 constitutes the third phase of HUGS development. The goal of HUGS-3 is to create a next-generation system that addresses the seven areas of improvement identified from the use of HUGS-1 and HUGS-2 and analysis of the data they captured. Each area of improvement generated two or more high-level functional requirements. All decisions made in the design and development of HUGS-3 will trace their origin back to those requirements in the context of the key operations – recording infant grasp force and upper extremity movement – that, in turn, are central to the goal of identifying neuromotor delay early to allow optimal timing of therapeutic intervention.

Infant, family, and researcher experience gained through the two phases of HUGS development that have taken place since 2019 suggest that the seven areas identified for improvement in the HUGS series can be addressed by a wireless, sensorized toy interfaced to a smartphone app.

Area for Improvement	*Corresponding Requirements*	*Implications of Wireless Toy and Smartphone Interface for Satisfying Requirements*
Footprint	#1.1. HUGS-3 will be easy for families to put away when not in use.<br>#1.2. HUGS-3 will have a small footprint.	• The HUGS-3 hardware will consist of one or more toys small enough to fit into an infant's hand.<br>• The toys will be designed to conform with the HAI toy kit. (See area 6 and requirements.)<br>• The toy(s), together with a smartphone with an app to control them, could be small enough to fit into a drawer.
Complexity	#2.1. HUGS-3 will minimize complexity in setup.<br>#2.2. HUGS-3 will minimize complexity in operation.	• The toy(s) might activate with a switch on the unit itself.<br>• The smartphone app will guide all interactions with the sensorized toys.<br>• Design of the user interface on the app is a critical component of managing complexity.
Durability	#3.1. HUGS-3 will resist being pulled apart by the infant.<br>#3.2. HUGS-3 will be hardened against being dropped or thrown by the infant.	• Problems with the durability of HUGS-2 were largely a function of fragile materials used in 3D printing and solder joints put under stress.<br>• Materials and fabrication methods will be essential to developing a toy robust to infant (ab)use.
File Handling	#4.1. HUGS-3 will accommodate large file uploads with no data loss.<br>#4.2. HUGS-3 will have ample local storage to hedge problems with file upload.	• The burden of data upload will be associated with the feed from the commercial camera to be incorporated into the system.<br>• Since depth data was problematic in our prior analysis, we will focus on simple RGB formats.<br>• This requirement for camera upload from the smartphone will largely fall to Software for execution.

Area for Improvement	*Corresponding Requirements*	*Implications of Wireless Toy and Smartphone Interface for Satisfying Requirements*
		• The small force files are not expected to be a problem.<br>• OneDrive may or may not be an interface in HUGS-3 as it was in HUGS-2.
Force Read Accuracy	#5.1. HUGS-3 will measure force uniformly regardless of the position of the infant's hand during grasp.	• The HUGS-3 Summer 2022 protype featured a configuration using 3 FSRs mounted at approximate 45-degree angles so that grasp originating from any direction might be uniformly captured. This configuration is one possibility going forward.<br>• A bladder configuration (further debrief of Dr. Lum is needed) for distributing and accurately reporting force is another possibility.
Measuring Clinical Criteria	#6.1. HUGS-3 will allow free movement of the infant's hand and arm, in any direction, during grasp.<br>#6.2. HUGS-3 will capture infant grasp activity from the front.	• Untethered toys, modeled on the HAI toy set, are essential to free manipulation by infants.<br>• Size and weight, grasping surface affordance, will also be factors in meeting this requirement.
Stimulus	#7.1. HUGS-3 will provide auditory feedback to the infant during grasp.<br>#7.2. HUGS-3 will provide tactile feedback to the infant during grasp.	• Auditory and tactile feedback is a criterial for a toy to be included in the HAI kit.<br>• In HUGS-2, these feedback modes were provided electronically. They may be provided electronically or passive mechanically in HUGS-3 going forward.

HUGS-3 Operational View (Mission Level)

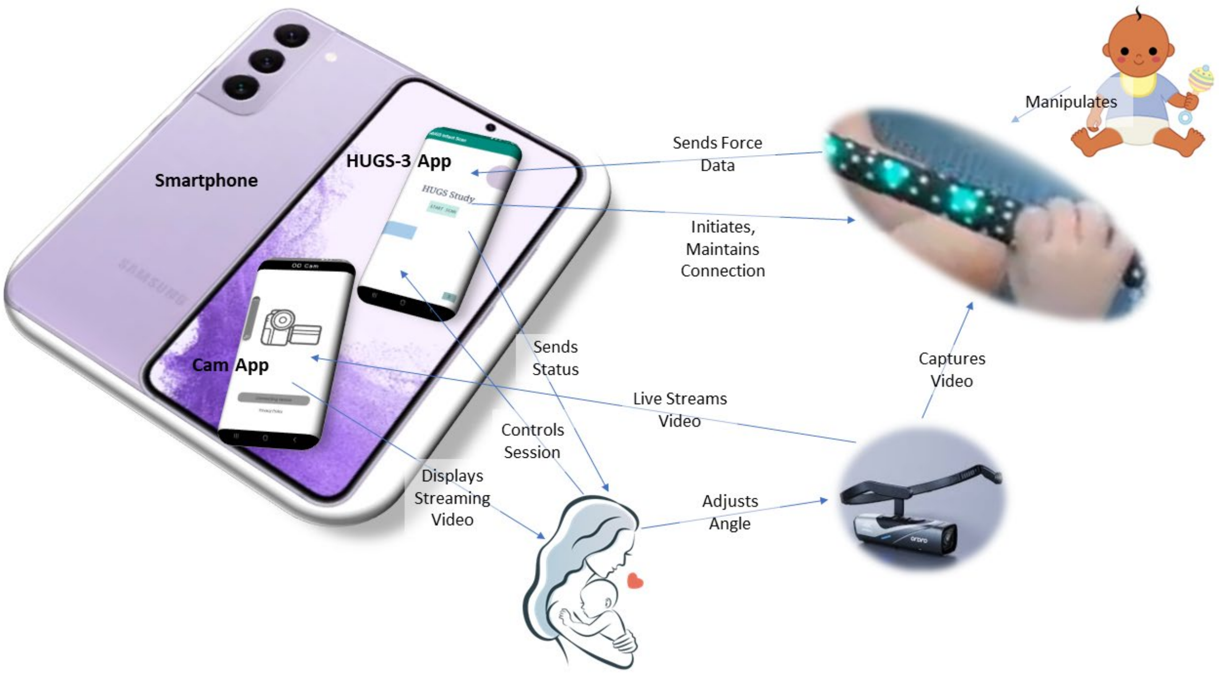

1. To record the forces generated by an infant’s grasp
2. To record infants’ hand and arm movements as they grasp objects
