# Supplementary material for: Evolution of a System to Monitor Infant Neuromotor Development in the Home: Lessons from COVID-19
Source: Healthcare (Basel). 2023 Mar 7;11(6):784. doi: 10.3390/healthcare11060784 (PMC10048217; doi:10.3390/healthcare11060784)
Supplement: Supplementary file 1 [file healthcare-11-00784-s001.zip › Supplement C - Grasp force-related outcomes.pdf]

Supplement C

Excerpt from Kuo, H.-H. (2023). *Home assessment of grasp development in infants for fine motor delay* [Dissertation]. The Catholic University of America.

Grasp force-related outcomes

The procedures developed for measuring grasp for in the HUGS-1 study were replicated for HUGS-2. The measures are reviewed below.

Grasp forces applied to the surface of the instrumented toy bar were sampled at 30 Hz, digitized, timestamped, converted to grams and written to the HUGS-2 system log. Grasp frequency and duration, peak grasp force, average grasp force, and the coefficient of variation (the standard deviation of the grasp force normalized by mean force, CV) were calculated in MATLAB 2020a [1]. Each qualified grasp event was coded as unimanual (force was exerted by only one hand) or bimanual (both hands were grasping the toy simultaneously). The location on the bar where the grasp took place was identified as inside, middle, or outside. Grasps that crossed the midline of the bar toy were also recorded. This coding was used to calculate the percentages of bimanual grasp, inside, middle, and outside bar grasps, and the midline crossing grasps for each two-minute trial. To assess asymmetry, right hand ratios (R-ratios) were calculated for each outcome by dividing the right-hand outcome by the result of the right-hand outcome plus the left-hand outcome.

Data analysis

The means/SD and confidence intervals were calculated based on the collected data. Plots were made for each outcome measure to compare the results of the infants at-risk to those of the TD group [3]. The grasp force-related outcome measures included peak force, mean force, accumulated grasping time, total number of grasps, and force CV for each grasp, and the R-ratio for the peak force, mean force, grasp frequency, and accumulated grasping time. The kinematic-

related outcome measures included average velocity and peak velocity of the left and right elbows and wrists, and the total path length of the left and right wrists.

A linear mixed effects model (LMM) was selected for data analysis to assess whether age showed a significant effect on each of the outcome measures above for the at-risk infants, using SPSS version 25.0 [2]. The LMM included participants as the random effect. Considering the very small sample size of this pilot study, the model only included age as the fixed effect. Age was treated as a continuous covariate. We used a random intercepts effect model. The "variance components" setting was used for the Covariance Type in SPSS. This setting assigns a scaled identity structure to each of the specified random effects. Dependent variables included in the analysis were the peak force, mean force, accumulated grasping time, total number of grasps, and force CV for each grasp. They also included the R-ratios for the peak force, mean force, grasping frequency, and accumulated grasping time and kinematic related outcomes including average velocity and peak velocity of both right and left elbows, and total path lengths of the left and right wrists.

To assess whether there were any significant differences in the changing trends of the outcome measures between the TD infant group and the at-risk infant group, we combined the data of the two groups and use a linear mixed effects model to test whether there were significant interaction effects between Age and Group. The model included participants as the random effect. Age was treated as a continuous covariate, and we used a random intercepts effect model. The same "variance components" setting was used for the Covariance Type in SPSS. To

Supplement C

Excerpt from Kuo, H.-H. (2023). *Home assessment of grasp development in infants for fine motor delay* [Dissertation]. The Catholic University of America.

facilitate maximal consideration of trends emerging from this exploratory study with a very small (n=5) sample size, the significance level was set at $\alpha = 0.1$.

RESULTS

A total of 651 recorded grasp events from five infants at risk for developmental delay were analyzed during this study. According to the results of the LMM, the following grasp related outcomes increased significantly with age. (Figure 3, y-axis variable codes are included in brackets for ease of cross-reference.)

- All grasp frequency [ALLFrequency] (slope = 2.710, $F(1, 21.122)=3.780$, $p = 0.065$)
- All accumulated grasping time [AllAccumuTime] (slope = 11.623, $F(1, 21.739)=3.110$, $p = 0.092$)
- Peak grasp force [AH_PeakForce] (slope = 90.521, $F(1, 20.300)=3.594$, $p = 0.072$)
- Average grasp force [AH_meanForce] (slope = 47.803, $F(1, 19.41)=3.110$, $p = 0.008$)
- The percentage of bi-manual grasps [BH_frequentPercent] (slope = 0.058, $F(1, 20.960)=14.192$, $p = 0.001$)
- The percentage of grasps at the inside of the bar toy [InsidePerc] (slope = 0.034, $F(1, 19.044)=4.319$, $p = 0.051$)

Only the force CV [CV] (slope = -0.033, $F(1, 21.705)=3.785$, $p = 0.065$) and the percentage of grasps at the middle part of the bar toy [MiddlePerc] (slope = -0.033, F(1,

20.428)=3.817, p = 0.065) showed a significant *decreasing* trend as infants' age increased. Age didn't show any significant effect on the percentage of grasps at the end side of the bar toy, on the percentage of grasps crossing the midline, or on the R-ratios for grasp frequency. Statistical results are listed below:

- The percentage of grasps at the end side of the bar toy [OutsidePerc] (slope = 0.000, F(1, 22)=0.001, p = 0.979)
- The percentage of grasps crossing the midline [not graphed] (slope = -0.002, F(1, 22)=0.424, p = 0.522)
- The R-ratios for grasp frequency [Rratio_frequency] (slope = 0.017, F(1, 22)=0.616, p = 0.441)

Among the tests for the interaction effect of Age and Group on both TD and the at-risk infants, only the average grasp force [AH_meanForce] (slope = 26.265, F(1, 76.405)=3.564, p = 0.063) showed a significant interaction effect of Age and Group, indicating that as age increased, the average grasp force of the at-risk group increased significantly faster than did that of the TD group. For all the other grasp-related outcomes, there was no significant interaction effect of Age and Group, showing that as age increased, there was no significant difference in the changing trends of the other outcomes between the infants in the TD group and the infants in the at-risk group. The Group factor didn't show any significant effect on any of the grasp-related outcomes between two the groups, indicating that no significant differences between groups were found in the grasp related outcomes.

Supplement C

Excerpt from Kuo, H.-H. (2023). *Home assessment of grasp development in infants for fine motor delay* [Dissertation]. The Catholic University of America.

Figure 3 below shows the comparison of the outcomes for each at-risk infant juxtaposed against those calculated for infants in the TD group. Table 3 shows the between-subjects means and standard deviations for all outcome measures at different ages for all at-risk and TD infants in the study, organized by month.

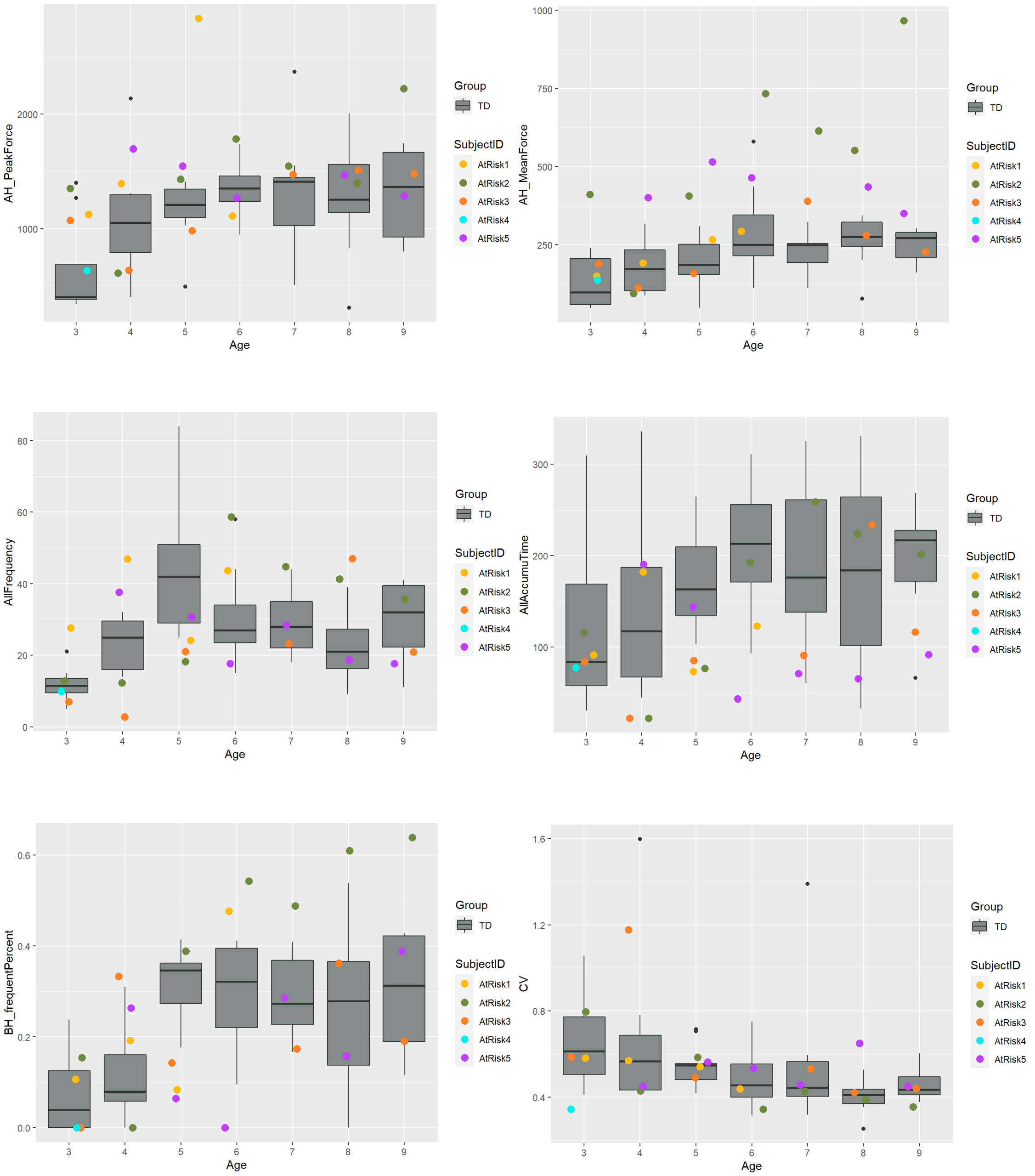

Supplement C

Excerpt from Kuo, H.-H. (2023). *Home assessment of grasp development in infants for fine motor delay* [Dissertation]. The Catholic University of America.

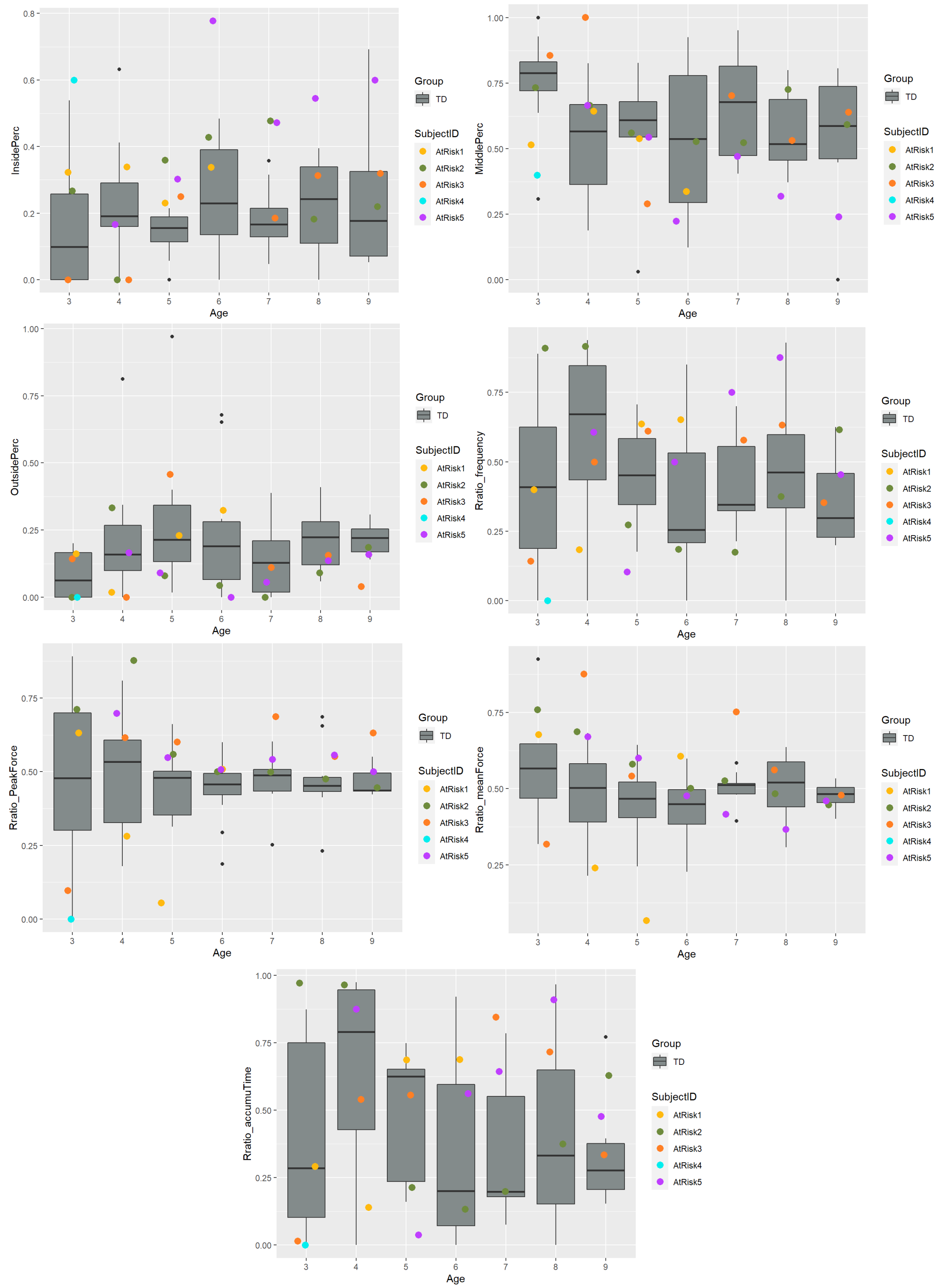

Supplement C

Excerpt from Kuo, H.-H. (2023). *Home assessment of grasp development in infants for fine motor delay* [Dissertation]. The Catholic University of America.

Figure 3. The grasp related outcome comparisons between each at-risk infant and the infants in the TD group.

Table 3. Between-subjects means, standard deviations for all outcome measures at different age in months for the at-risk infants' group

		**3 Months**		**4 Months**		**5 Months**		**6 Months**		**7 Months**		**8 Months**		**9 Months**	
		Mean	Std.	Mean	Std.	Mean	Std.	Mean	Std.	Mean	Std.	Mean	Std.	Mean	Std.
Peak Force	TD	633.15	408.28	1075.01	512.03	1159.75	266.23	1336.79	210.74	1294.90	508.91	1260.79	445.35	1303.48	387.10
	At-Risk	1045.18	260.58	1083.79	474.13	1320.08	691.14	1388.22	287.82	1510.27	37.92	1458.09	48.48	1662.79	404.31
Mean Force	TD	128.82	77.08	181.56	83.47	189.28	76.25	285.68	129.97	234.59	81.72	264.30	75.96	248.75	53.03
	At-Risk	222.27	110.95	200.22	121.91	360.39	135.37	497.97	181.66	502.28	112.47	422.42	111.54	514.72	323.11
CV	TD	0.66	0.20	0.68	0.37	0.55	0.10	0.50	0.14	0.55	0.31	0.41	0.07	0.46	0.08
	At-Risk	0.58	0.16	0.66	0.31	0.55	0.03	0.44	0.08	0.48	0.05	0.49	0.12	0.42	0.04
Grasp Frequency	TD	11.88	4.54	23.50	6.93	45.78	20.96	30.25	11.14	30.22	9.33	22.80	9.42	29.50	11.12
	At-Risk	14.50	8.08	25.00	18.07	23.33	4.82	40.33	16.94	34.00	11.00	35.67	12.04	25.00	7.87
Bi-manual Percent	TD	0.08	0.09	0.12	0.10	0.32	0.07	0.29	0.11	0.30	0.09	0.26	0.16	0.30	0.13
	At-Risk	0.07	0.07	0.20	0.12	0.20	0.13	0.34	0.24	0.33	0.16	0.38	0.18	0.41	0.18
Accumulated Grasp Time	TD	127.77	97.80	147.22	98.45	174.47	50.98	213.81	63.65	182.49	86.85	180.67	97.63	192.94	65.24
	At-Risk	92.26	14.59	104.23	82.22	101.90	28.73	119.77	61.11	174.93	84.03	174.51	77.31	136.68	46.87
Grasp Inside Bar Percent	TD	0.17	0.19	0.25	0.18	0.14	0.07	0.24	0.16	0.19	0.09	0.22	0.13	0.25	0.23
	At-Risk	0.30	0.21	0.13	0.14	0.30	0.05	0.51	0.19	0.33	0.15	0.35	0.15	0.38	0.16
Grasp Middle Bar Percent	TD	0.75	0.20	0.52	0.21	0.58	0.21	0.53	0.29	0.67	0.18	0.56	0.14	0.53	0.27
	At-Risk	0.63	0.18	0.74	0.15	0.47	0.11	0.36	0.13	0.61	0.09	0.53	0.17	0.49	0.18
Grasp end Bar Percent	TD	0.08	0.09	0.23	0.24	0.29	0.27	0.23	0.22	0.14	0.12	0.22	0.11	0.22	0.06
	At-Risk	0.08	0.08	0.13	0.13	0.21	0.15	0.12	0.14	0.06	0.06	0.13	0.03	0.13	0.06
Mid-Crossing Percent	TD	0.00	0.00	0.00	0.00	0.01	0.02	0.01	0.01	0.00	0.00	0.04	0.07	0.05	0.08
	At-Risk	0.03	0.06	0.00	0.00	0.00	0.00	0.00	0.00	0.01	0.01	0.00	0.00	0.01	0.02
R ratio_ frequency	TD	0.41	0.30	0.60	0.32	0.46	0.17	0.35	0.25	0.43	0.17	0.49	0.27	0.36	0.16
	At-Risk	0.36	0.35	0.55	0.26	0.33	0.23	0.45	0.19	0.38	0.20	0.63	0.20	0.47	0.11

Supplement C

Excerpt from Kuo, H.-H. (2023). *Home assessment of grasp development in infants for fine motor delay* [Dissertation]. The Catholic University of America.

R ratio_ Peak Force	TD	0.46	0.31	0.50	0.21	0.46	0.12	0.44	0.10	0.47	0.09	0.47	0.12	0.47	0.05
	At-Risk	0.36	0.31	0.62	0.22	0.57	0.22	0.51	0.00	0.59	0.09	0.53	0.04	0.53	0.08
R ratio_ Mean Force	TD	0.58	0.19	0.48	0.16	0.45	0.11	0.43	0.10	0.50	0.05	0.50	0.10	0.48	0.04
	At-Risk	0.59	0.19	0.62	0.23	0.57	0.22	0.53	0.06	0.64	0.11	0.47	0.08	0.46	0.01
R ratio_ accumulate Time	TD	0.39	0.35	0.65	0.34	0.51	0.23	0.33	0.31	0.35	0.23	0.41	0.32	0.34	0.21
	At-Risk	0.32	0.39	0.63	0.32	0.27	0.26	0.46	0.24	0.52	0.32	0.67	0.22	0.48	0.12

Supplement C

Excerpt from Kuo, H.-H. (2023). *Home assessment of grasp development in infants for fine motor delay* [Dissertation]. The Catholic University of America.

REFERENCES

[1] *MATLAB*. 2020 [cited 2020 December 29, 2020]; R2020a:[Available from: https://www.mathworks.com/products/new_products/release2020a.html.

[2] West, B.T., Analyzing longitudinal data with the linear mixed model's procedure in SPSS. Evaluation & the Health Professions, 2009. 32(3): p. 207-228.

[3] Kuo, H., Wang, J., Schladen, M. M., Chang, T., Morozova, O. M., Croce, U. D., Kukke, S. N., & Lum, P. S. (2022). Hand Use and Grasp Sensor System in Monitoring Infant Fine Motor Development. Archives of Rehabilitation Research and Clinical Translation, 4(3), 100203. https://doi.org/10.1016/j.arrct.2022.100203
