# Supplementary material for: Evolution of a System to Monitor Infant Neuromotor Development in the Home: Lessons from COVID-19
Source: Healthcare (Basel). 2023 Mar 7;11(6):784. doi: 10.3390/healthcare11060784 (PMC10048217; doi:10.3390/healthcare11060784)
Supplement: Supplementary file 1 [file healthcare-11-00784-s001.zip › Supplement D - Kinematic outcomes.pdf]

Supplment D

Excerpt from: Kuo, H.-H. (2023). *Home assessment of grasp development in infants for fine motor delay* [Dissertation]. The Catholic University of America.

Kinematic-related results from the spontaneous movements videos

A total of 25, 3D-camera recorded spontaneous movements videos from five infants at risk for developmental delay were analyzed during this study. The following kinematic parameters were calculated in MATLAB 2020a.[1] from the X, Y, Z values of the 3D points of the tracked[1] joints: a) average velocity and peak velocity of the elbows and wrists, on both the left and right sides; and b) total path length of the left wrist and the right wrist. A linear mixed effects model (LMM) was selected for data analysis to assess whether age showed a significant effect on each of the outcome measures above for the at-risk infants, using SPSS version 25.0 [2]. The LMM included participants as the random effect. Considering the very small sample size of this pilot study, the model only included age as the fixed effect. Age was treated as a continuous covariate. We used a random intercepts effect model. The "variance components" setting was used for the Covariance Type in SPSS. This setting assigns a scaled identity structure to each of the specified random effects. Dependent variables included in the analysis were average velocity and peak velocity of both right and left elbows, and total path lengths of the left and right wrists.

To assess whether there were any significant differences in the changing trends of the outcome measures between the TD infant group and the at-risk infant group, we combined the data of the two groups and use a linear mixed effects model to test whether there were significant interaction effects between Age and Group. The model included participants as the random effect.

[1] Tracking was performed as described in Balta D, Kuo H, Wang J, et al. Characterization of Infants' General Movements Using a Commercial RGB-Depth Sensor and a Deep Neural Network Tracking Processing Tool: An Exploratory Study. Sensors (Basel). 2022;22(19):7426. doi:10.3390/s22197426

Supplment D

Excerpt from: Kuo, H.-H. (2023). *Home assessment of grasp development in infants for fine motor delay* [Dissertation]. The Catholic University of America.

Age was treated as a continuous covariate, and we used a random intercepts effect model. The same "variance components" setting was used for the Covariance Type in SPSS. To facilitate maximal consideration of trends emerging from this exploratory study with a very small (n=5) sample size, the significance level was set at $\alpha = 0.1$.

According to the results of the LMM, all the following kinematics parameters increased significantly with age: the average velocity, peak velocity, the total moving path lengths of both the left and right elbows and the wrists.

- Left elbow velocity (slope = 0.007, $F(1, 21.044)=10.377$, $p = 0.004$)
- Left wrist velocity (slope = 0.062, $F(1, 21.044)=3.899$, $p = 0.062$)
- Right elbow velocity (slope = 0.007, $F(1, 21.158)=8.885$, $p = 0.007$)
- Right wrist velocity (slope = 0.008, $F(1, 23)=6.195$, $p = 0.020$)
- Left elbow peak velocity (slope = 0.063, $F(1, 23)=10.507$, $p = 0.004$)
- Left wrist peak velocity (slope = 0.069, $F(1, 23)=7.470$, $p = 0.012$)
- Right elbow peak velocity (slope = 0.063, $F(1, 23)=4.355$, $p = 0.048$)
- Right wrist peak velocity (slope = 0.056, $F(1, 23)=1.985$, $p = 0.059$)
- Left elbow total path length (slope = 1.774, $F(1, 20.719)=14.838$, $p <0.001$)
- Left wrist total path length (slope = 1.458, $F(1, 20.900)=6.719$, $p =0.017$)
- Right elbow total path length (slope = 1.825, $F(1, 20.808)=12.051$, $p =0.002$)
- Right wrist total path length (slope = 1.866, $F(1, 21.002)=11.880$, $p =0.002$)

Supplment D

Excerpt from: Kuo, H.-H. (2023). *Home assessment of grasp development in infants for fine motor delay* [Dissertation]. The Catholic University of America.

Age did not show a significant effect on the CV of the velocity or the total moving duration. The statistic result is listed below:

- CV of the left elbow average velocity (slope = 0.074, F(1, 45.843)=0.204, p =0.654)
- CV of the left wrist average velocity (slope = 0.076, F(1, 45808)=1.999, p =0.186)
- CV of the right elbow average velocity (slope = 0.146, F(1, 46.716)=4.517, p =0.039)
- CV of the right wrist average velocity (slope = 0.044, F(1, 46.808)=0.525, p =0.472)
- Left elbow move duration (slope = -1.664, F(1, 42.053)=0.026, p =0.873)
- Left wrist move duration (slope = -1.664, F(1, 42.053)=0.026, p =0.873)
- Right elbow move duration (slope = 10.499, F(1, 40.163)=1.255, p =0.269)
- Right wrist move duration (slope = 10.441, F(1, 40.194)=1.232, p =0.274)

When comparing the changing trends of these kinematic parameters between the TD and at-risk groups, there was no significant interaction effect of Age and Group found in any of these kinematics parameters. No significant slope differences were found in the increasing trends of kinematics during the spontaneous movements between the at-risk infants and the TD infants. However, the Group factor did show a significant effect on the following outcome measures, which means that for the at-risk infants, the average velocity of the right elbow, the moving duration of the right elbow and wrist, and the total path lengths of both elbows and the right wrist were

Supplment D

Excerpt from: Kuo, H.-H. (2023). *Home assessment of grasp development in infants for fine motor delay* [Dissertation]. The Catholic University of America.

significantly bigger than that of the TD group (Table 1, Figure 1). Figure 1 shows the comparison of the outcomes between each at-risk infant and the TD group.

- Right elbow velocity (slope = 0.035, $F(1, 46.716)=3.110$, $p=0.084$)
- Right elbow moving duration (slope = 63.450, $F(1, 44.048)=3.775$, $p=0.058$)
- Right wrist moving duration (slope = 63.172, $F(1, 44.074)=3.719$, $p=0.060$)
- Left elbow total path length (slope = 9.756, $F(1, 46.661)=4.055$, $p=0.050$)
- Right elbow total path length (slope = 13.943, $F(1, 46.548)=10.056$, $p=0.003$)
- Right wrist total path length (slope = 11.671, $F(1, 38.469)=3.275$, $p=0.078$)

Table 1. Between-subjects means and standard deviations for all kinematic outcome measures in spontaneous videos at different age in months for At-Risk infants' group

		3 Months		4 Months		5 Months		6 Months		7 Months		8 Months		9 Months	
	Group	Mean	Std	Mean	Std	Mean	Std	Mean	Std	Mean	Std	Mean	Std	Mean	Std
LE_Velocity	TD	0.04	0.02	0.06	0.03	0.04	0.03	0.05	0.02	0.06	0.01	0.06	0.00	NA	NA
	At-Risk	0.07	0.01	0.07	0.01	0.08	0.03	0.11	0.03	0.10	0.02	0.09	0.02	0.11	0.01
LW_Velocity	TD	0.07	0.03	0.08	0.02	0.07	0.06	0.08	0.00	0.11	0.01	0.07	0.00	NA	NA
	At-Risk	0.10	0.01	0.09	0.04	0.09	0.03	0.12	0.03	0.12	0.03	0.11	0.03	0.13	0.02
RE_Velocity	TD	0.03	0.02	0.05	0.01	0.05	0.02	0.04	0.01	0.07	0.02	0.06	0.00	NA	NA
	At-Risk	0.07	0.02	0.08	0.02	0.07	0.03	0.10	0.03	0.11	0.04	0.11	0.02	0.11	0.02
RW_Velocity	TD	0.06	0.03	0.07	0.03	0.08	0.05	0.06	0.00	0.11	0.02	0.07	0.00	NA	NA
	At-Risk	0.10	0.02	0.09	0.03	0.10	0.03	0.10	0.02	0.14	0.05	0.14	0.02	0.13	0.02
CV_LE_ Velocity	TD	0.71	0.25	0.88	0.48	0.67	0.24	0.79	0.04	1.14	0.08	0.80	0.00	NA	NA
	At-Risk	0.68	0.28	0.54	0.07	0.78	0.14	0.78	0.25	0.71	0.36	0.77	0.16	0.87	0.18
CV_LW_ Velocity	TD	0.99	0.20	0.94	0.12	0.85	0.28	1.08	0.04	1.17	0.08	1.13	0.00	NA	NA
	At-Risk	0.90	0.08	0.72	0.06	0.85	0.10	0.85	0.27	1.04	0.24	0.89	0.14	0.89	0.17
CV_RE_ Velocity	TD	0.63	0.26	0.71	0.32	0.79	0.20	0.86	0.28	1.08	0.13	0.82	0.00	NA	NA
	At-Risk	0.65	0.30	0.62	0.11	0.83	0.09	0.76	0.13	0.89	0.38	0.84	0.12	0.75	0.25
CV_RW_ Velocity	TD	0.94	0.23	0.87	0.21	0.96	0.18	1.05	0.01	1.06	0.10	0.96	0.00	NA	NA
	At-Risk	0.87	0.24	0.89	0.07	0.77	0.11	0.81	0.12	1.15	0.34	0.94	0.18	0.82	0.33
LE_peak_ Velocity	TD	0.20	0.20	0.50	0.54	0.21	0.21	0.32	0.19	0.54	0.10	0.40	0.00	NA	NA
	At-Risk	0.38	0.17	0.40	0.05	0.57	0.22	0.61	0.17	0.58	0.46	0.72	0.13	0.76	0.05

Supplment D

Excerpt from: Kuo, H.-H. (2023). *Home assessment of grasp development in infants for fine motor delay* [Dissertation]. The Catholic University of America.

LW_peak_Velocity	TD	0.47	0.41	0.71	0.15	0.42	0.59	0.78	0.06	0.76	0.27	0.67	0.00	NA	NA
	At-Risk	0.72	0.12	0.56	0.13	0.70	0.18	0.83	0.27	0.96	0.38	0.92	0.31	0.99	0.28
RE_peak_Velocity	TD	0.16	0.24	0.34	0.23	0.27	0.20	0.30	0.23	0.57	0.23	0.43	0.00	NA	NA
	At-Risk	0.48	0.21	0.46	0.17	0.49	0.25	0.59	0.18	0.77	0.55	0.81	0.15	0.66	0.30
RW_peak_Velocity	TD	0.40	0.44	0.46	0.26	0.52	0.46	0.57	0.07	0.82	0.21	0.56	0.00	NA	NA
	At-Risk	0.65	0.18	0.77	0.11	0.59	0.17	0.78	0.08	1.11	0.60	0.98	0.18	0.83	0.32
LE_move_duration	TD	100.26	54.88	134.40	36.86	65.43	46.85	93.34	26.98	135.83	14.76	63.19	0.00	NA	NA
	At-Risk	158.17	19.29	175.50	3.62	147.73	40.91	155.93	22.53	179.97	0.03	162.81	11.49	173.74	6.44
LW_move_duration	TD	100.26	54.88	134.40	36.86	65.43	46.85	93.34	26.98	135.83	14.76	63.19	0.00	NA	NA
	At-Risk	158.17	19.29	175.50	3.62	147.73	40.91	155.93	22.53	179.97	0.03	162.81	11.49	173.74	6.44
RE_move_duration	TD	84.10	59.89	131.09	38.78	83.26	42.92	90.60	10.00	149.50	9.42	113.24	0.00	NA	NA
	At-Risk	158.29	18.44	175.50	3.62	147.73	40.91	156.79	21.57	170.79	8.91	167.81	11.48	173.49	6.39
RW_move_duration	TD	84.10	59.89	131.79	39.36	83.26	42.92	90.60	10.00	149.50	9.42	113.24	0.00	NA	NA
	At-Risk	158.29	18.44	175.50	3.62	147.73	40.91	156.79	21.57	170.79	8.91	167.81	11.48	173.49	6.39
LE_total_path_length	TD	3.50	4.65	8.67	5.61	5.81	5.07	5.66	3.80	8.97	2.78	11.67	0.00	NA	NA
	At-Risk	17.88	1.93	21.09	3.41	22.63	7.79	29.88	8.08	26.57	1.18	27.08	6.66	28.14	2.97
LW_total_path_length	TD	8.05	7.33	12.12	5.20	9.33	10.56	8.73	1.50	15.53	3.35	10.71	0.00	NA	NA
	At-Risk	24.58	1.56	24.57	8.52	23.72	7.46	27.86	7.75	28.29	4.24	28.44	6.53	32.88	5.25
RE_total_path_length	TD	3.35	4.79	6.70	1.87	6.76	2.91	4.97	1.22	10.98	3.90	10.99	0.00	NA	NA
	At-Risk	19.98	3.23	22.92	5.22	18.79	7.14	26.81	8.71	26.72	4.49	30.71	3.81	28.52	4.60
RW_total_path_length	TD	6.70	7.74	9.83	5.83	11.49	10.82	8.58	1.24	17.96	3.81	12.17	0.00	NA	NA
	At-Risk	24.89	3.16	23.77	6.39	25.81	4.47	25.68	6.69	32.26	7.76	35.96	3.52	32.01	4.73

Supplment D

Excerpt from: Kuo, H.-H. (2023). *Home assessment of grasp development in infants for fine motor delay* [Dissertation]. The Catholic University of America.

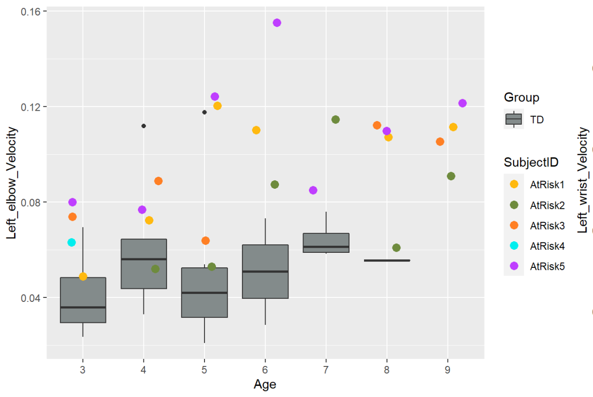

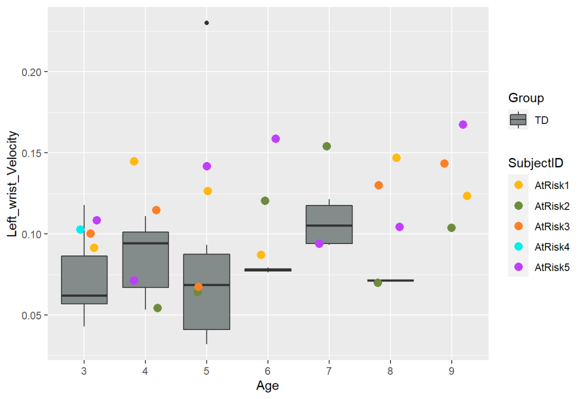

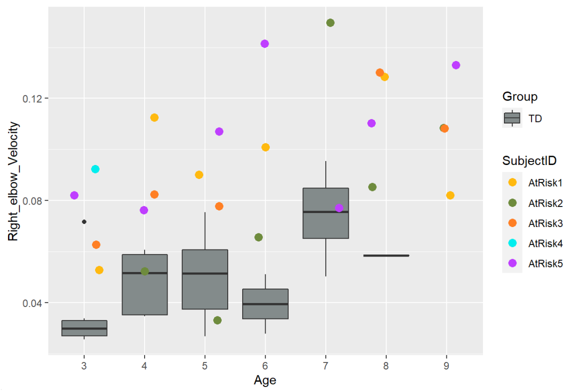

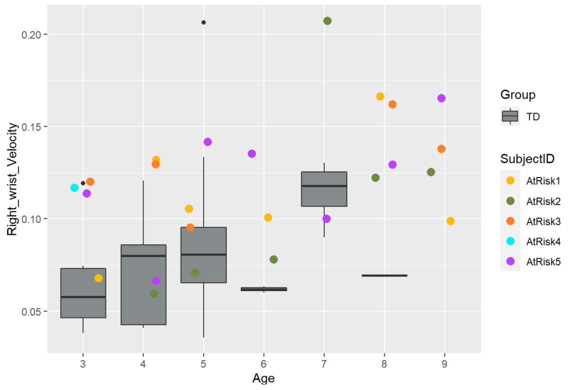

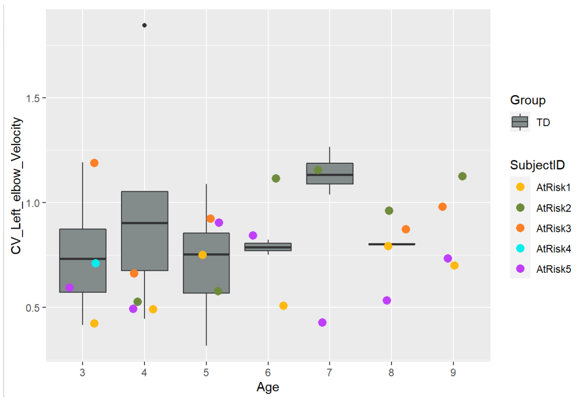

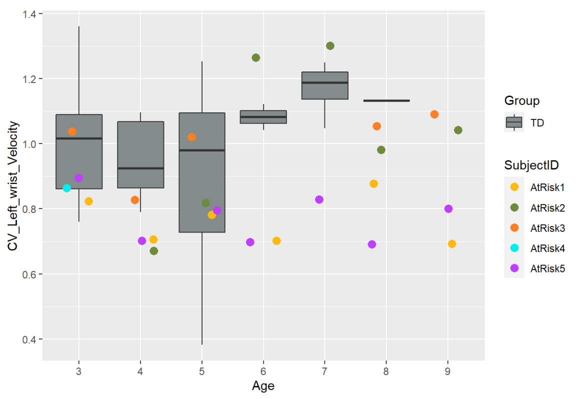

Supplment D

Excerpt from: Kuo, H.-H. (2023). *Home assessment of grasp development in infants for fine motor delay* [Dissertation]. The Catholic University of America.

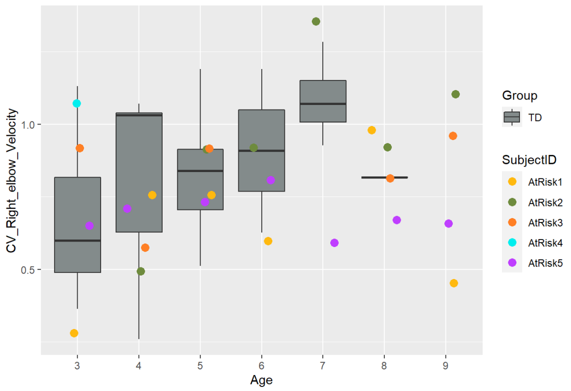

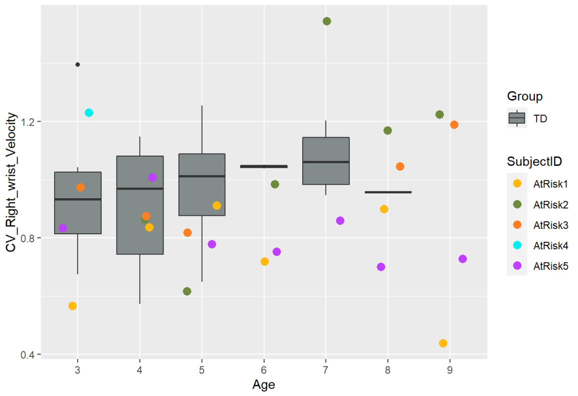

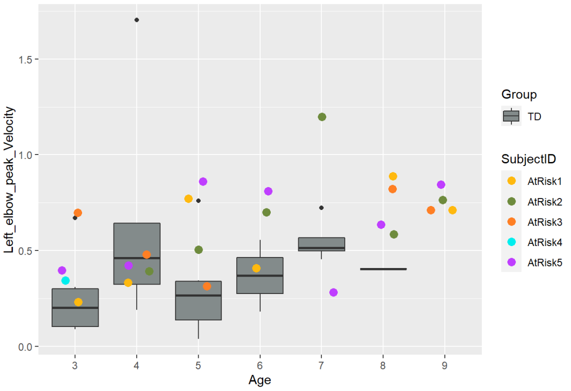

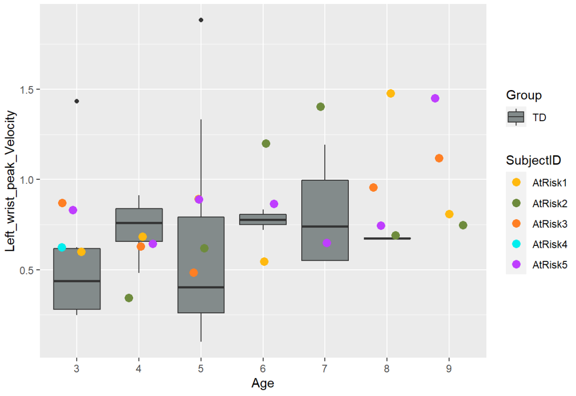

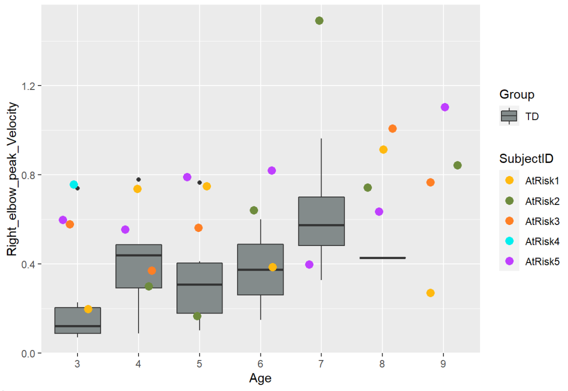

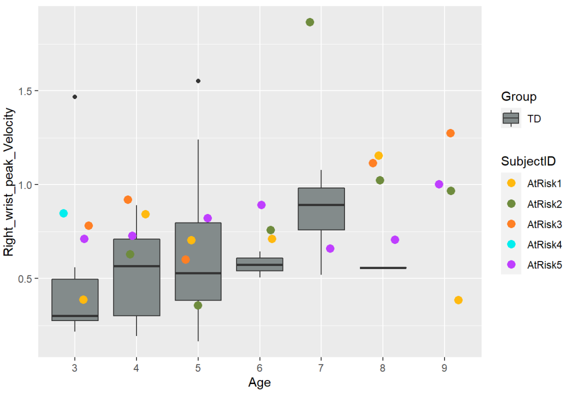

Supplment D

Excerpt from: Kuo, H.-H. (2023). *Home assessment of grasp development in infants for fine motor delay* [Dissertation]. The Catholic University of America.

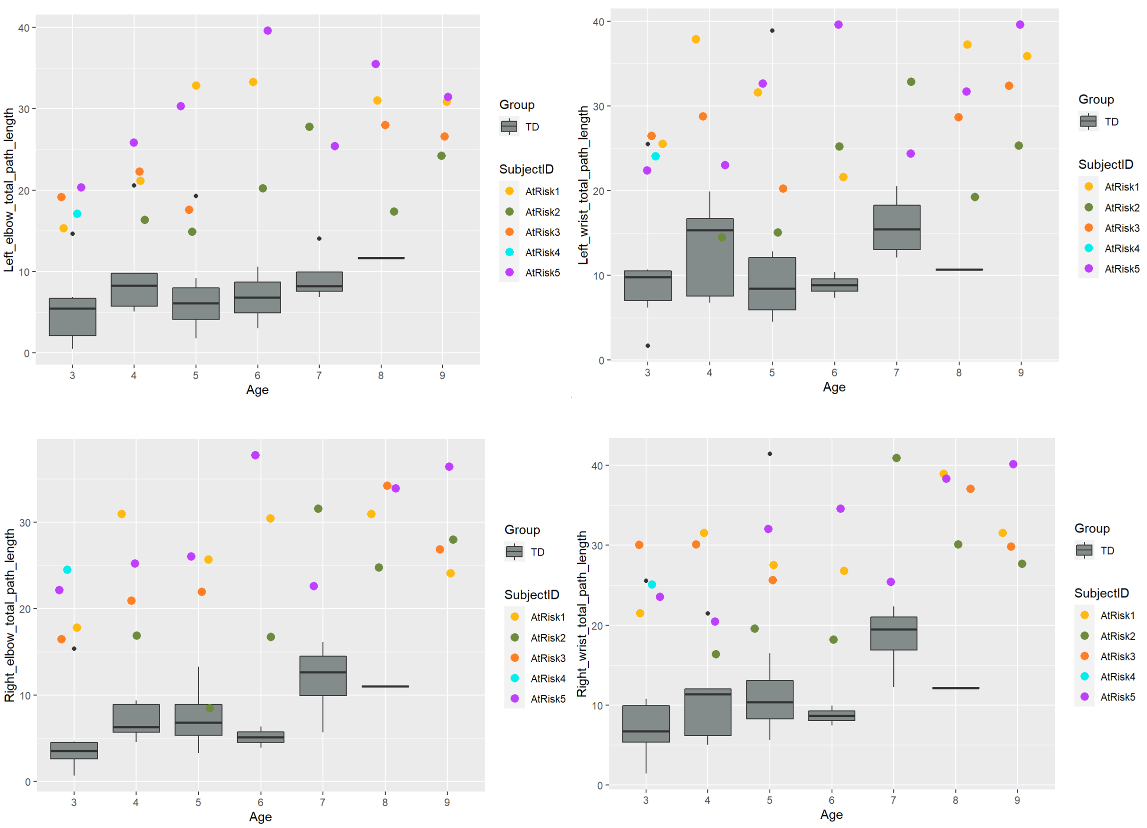

Figure 1. The kinematic parameter comparisons between each at-risk infant and the infants in the TD group during spontaneous movement.

Supplment D

Excerpt from: Kuo, H.-H. (2023). *Home assessment of grasp development in infants for fine motor delay* [Dissertation]. The Catholic University of America.

Reference

1. K Kuo H, Wang J, Schladen MM, et al. Hand Use and Grasp Sensor System in Monitoring Infant Fine Motor Development. *Arch Rehabil Res Clin Transl*. 2022;4(3):100203. doi:10.1016/j.arrct.2022.100203
