# Supplementary material for: Evolution of a System to Monitor Infant Neuromotor Development in the Home: Lessons from COVID-19
Source: Healthcare (Basel). 2023 Mar 7;11(6):784. doi: 10.3390/healthcare11060784 (PMC10048217; doi:10.3390/healthcare11060784)
Supplement: Supplementary file 1 [file healthcare-11-00784-s001.zip › Supplement E - HUGS-1 Kinamatics Extraction and Analysis Methods.pdf]

# Supplement E

Excerpt from: Kuo, H.-H. (2023). *Home assessment of grasp development in infants for fine motor delay* [Dissertation]. The Catholic University of America.

## METHODS: INFANT-IN-HOME MOVEMENT ASSESSMENT

### *Participants*

Fourteen infants participated in a longitudinal, in-home trial of a Hand Use and Grasp Sensor (HUGS) system designed to measure infant fine motor development in home environments. Criteria for inclusion included the following: 1) the infant had a gestational age between 37-42 weeks; 2) no complications were recorded during the mother's pregnancy and delivery; 3) the infant's age was between 12-36 weeks old and the parents were over 18 years of age. Infants with any known neurological conditions provided by parents' reports were excluded from the study.

Two infants dropped out during the study. At the start of the study, eight infants were three months old, one was five months old, and the remaining three were six months old. Ten testing sessions planned across six of the participants were curtailed by the Institutional Review Board due to risks associated with the COVID-19 pandemic that emerged in the later months of the study. In all, a total of 29 video testing sessions were available for analysis (Figure 1).

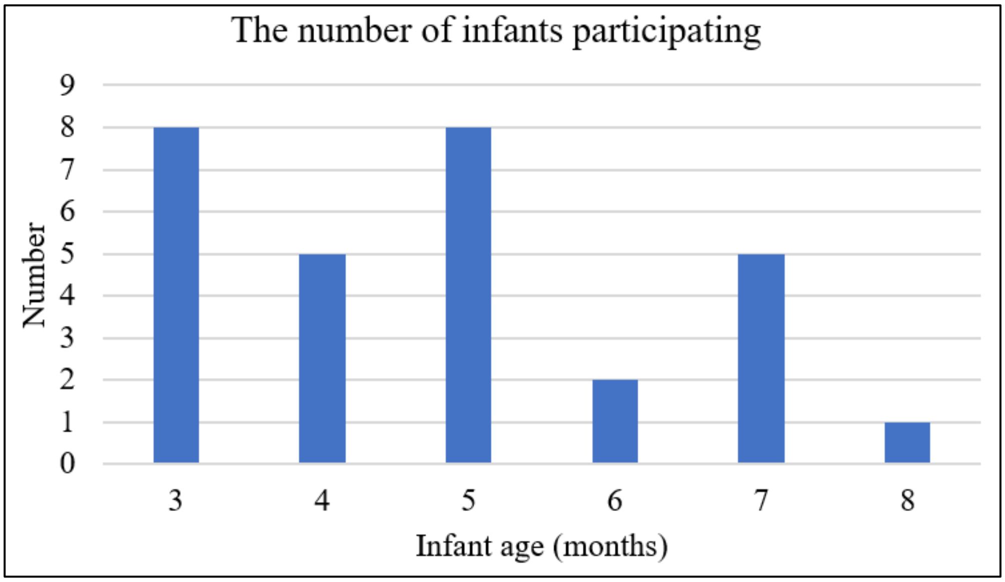

# Supplement E

Excerpt from: Kuo, H.-H. (2023). *Home assessment of grasp development in infants for fine motor delay* [Dissertation]. The Catholic University of America.

Figure 1. The number and ages of infants participating.

Table 1. Participants and Datapoints

| Subject ID | Gender | Age (months) | | | | | | |
|---|---|---|---|---|---|---|---|---|
| | | 3 | 4 | 5 | 6 | 7 | 8 | 9 |
| P01 | F | × | | × | | × | | |
| P02 | F | | | × | | | | |
| P03 | F | | | | | | | |
| P04 | M | | | × | × | | | |
| P05 | F | | | × | | × | × | |
| P06 | M | × | | × | × | × | | |
| P07 | F | × | × | × | | | | |
| P08 | F | × | × | × | | | | |
| P09 | F | × | × | | | × | | |
| P10 | F | × | × | × | | | | |
| P11 | F | × | | | | × | | |
| P12 | M | | | | | | | |
| P13 | M | | × | | | | | |
| P14 | M | × | | | | | | |

Abbreviations: F, female; M, male

## 3.4.2 Procedure

Researchers made home visits monthly to participating families to collect video of infant spontaneous upper extremity movement concurrent with the collection of HUGS grasp data (See Chapter 2). During each recording of spontaneous movement, the infant was seated in a standardized infant seat in semi-recline. Once infants were securely seated, we started the camera and recorded three minutes of spontaneous upper limb movement.

## 3.4.3 3D points based infant kinematic analysis

We identified infants' upper extremity PoI using DeepLabCut and integrated the RealSense depth sensor data as described in Balta et al., 2022 [1]. We performed motion analysis on each infant's elbow and wrist, including average speed, maximum speed, duration, and moving distance,

as recorded during the three-minute 3D video. The following kinematic parameters were calculated in MATLAB 2020a [2] from the X, Y, Z values of the 3D points of the infants' tracked joints: 1.) average velocity and peak velocity of both left and right elbows and wrists; 2.) the duration of movement of the left and right arms and 3.) the total path length of the left and right wrists.

3.4.4 Statistical Analysis

To examine whether age showed a significant effect on each of the kinematic parameters, a linear mixed effects model (LMM) was selected for data analysis and calculated using SPSS version 25.0 [3]. The model included participants as the random effect. Considering the very small sample size of this pilot study, the model included only age as the fixed effect. Age was treated as a continuous covariate. We used a random intercepts effect model. The "variance components" setting was used for the Covariance Type in SPSS, which assigns a scaled identity structure to each of the specified random effects. Dependent variables included kinematic related outcomes: average velocity, peak velocity, total moving duration, and total moving paths of elbows and wrists on the left and right sides.

3.5 RESULTS: INFANT IN-HOME MOVEMENT ASSESSMENT

The results of the LMM showed that age had a significant effect on the changing of the kinematic parameters listed below. When the infant participants' age increased, all the following kinematic parameters had significant increasing trends ($\alpha$=0.05).

- Right elbow average velocity (slope = 0.010, $F(1, 15.143)=45.029$, $p < 0.001$)
- Right wrist average velocity (slope = 0.011, $F(1, 14.374)=7.441$, $p = 0.016$)
- CV of the right elbow average velocity (slope = 0.076, $F(1, 24)=4.828$, $p = 0.038$)

# Supplement E

Excerpt from: Kuo, H.-H. (2023). *Home assessment of grasp development in infants for fine motor delay* [Dissertation]. The Catholic University of America.

- Right elbow peak velocity (slope = 0.090, $F(1, 18.914)=9.800$, $p=0.006$)
- Right elbow total path length (slope = 1.587, $F(1, 15.292)=28.554$, $p<0.001$)
- Right wrist total path length (slope = 1.860, $F(1, 14.121)=9.197$, $p=0.009$)

It is noteworthy that all the kinematic parameters above relate to the right side of the body. Only the following right-sided parameters did not increase significantly with infants' age:

- CV of the right wrist average velocity (slope = 0.025, $F(1, 24)=0.926$, $p=0.345$)
- Right wrist peak velocity (slope = 0.089, $F(1, 16.12)=4.211$, $p=0.057$)

Age did not show a significant effect on the relevant kinematic parameters of the left side of the body, including average velocity, peak velocity, total moving duration, and total moving path of the left elbow and the left wrist. The statistical results are listed below:

- Left elbow average velocity (slope = 0.003, $F(1, 24)=1.157$, $p=0.293$)
- Left wrist average velocity (slope = 0.006, $F(1, 13.937)=2.196$, $p=0.161$)
- CV of the left elbow average velocity (slope = 0.047, $F(1, 24)=1.138$, $p=0.297$)
- CV of the left wrist average velocity (slope = 0.040, $F(1, 23.929)=1.854$, $p=0.186$)
- Left elbow peak velocity (slope = 0.027, $F(1, 24)=0.358$, $p=0.555$)
- Left wrist peak velocity (slope = 0.043 $F(1, 23.998)=0.565$, $p=0.459$)
- Left elbow total path length (slope = 0.664, $F(1, 24)=0.968$, $p=0.335$)
- Left wrist total path length (slope = 0.5, $F(1, 14.282)=0.358$, $p=0.559$)

# Supplement E

Excerpt from: Kuo, H.-H. (2023). *Home assessment of grasp development in infants for fine motor delay* [Dissertation]. The Catholic University of America.

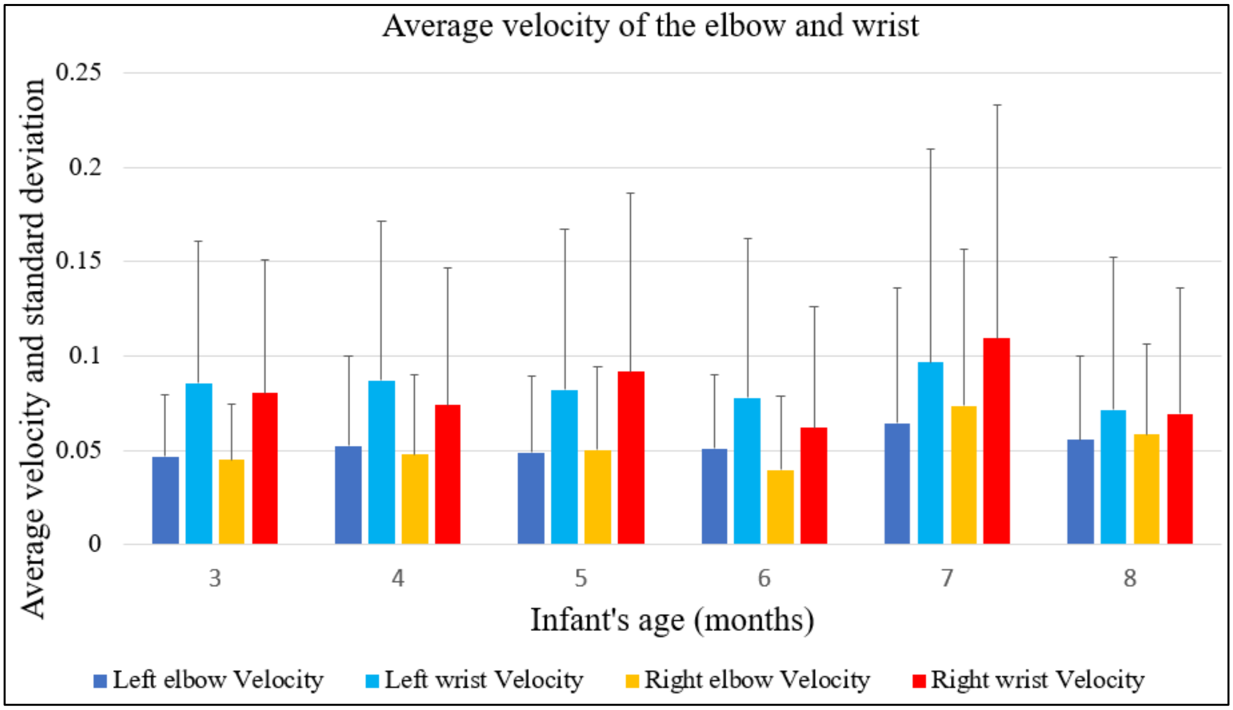


Figure 2. The calculated average velocity of the elbow and wrist in the state of spontaneous movement.

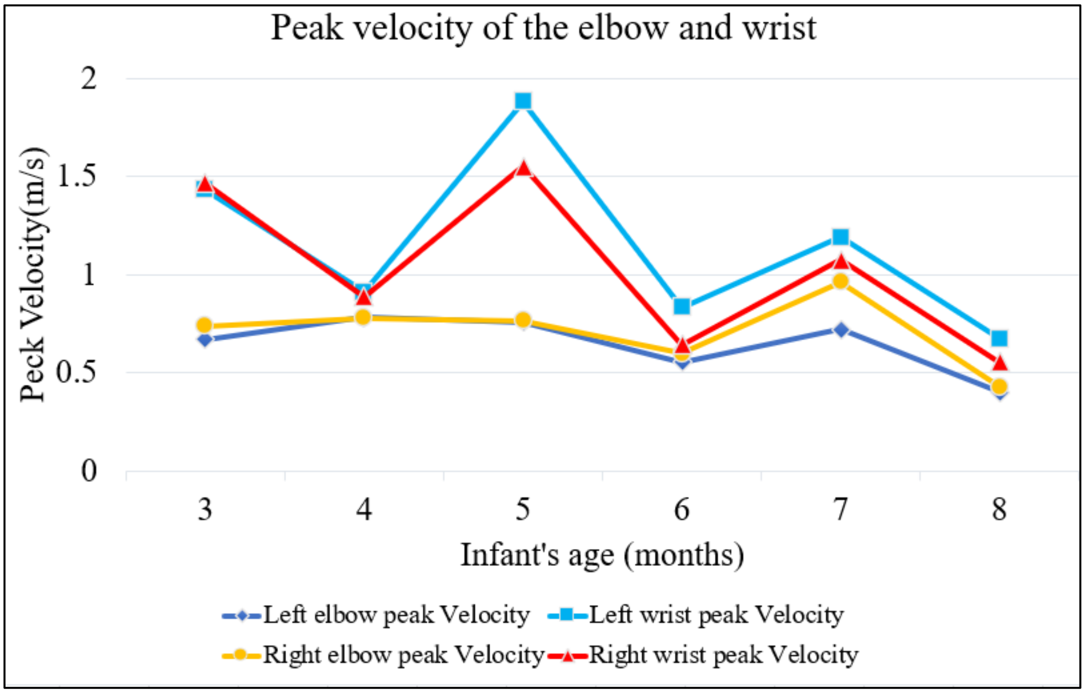


Figure 3. The calculated peak velocity of the elbow and wrist in the state of spontaneous movement.

# Supplement E

Excerpt from: Kuo, H.-H. (2023). *Home assessment of grasp development in infants for fine motor delay* [Dissertation]. The Catholic University of America.

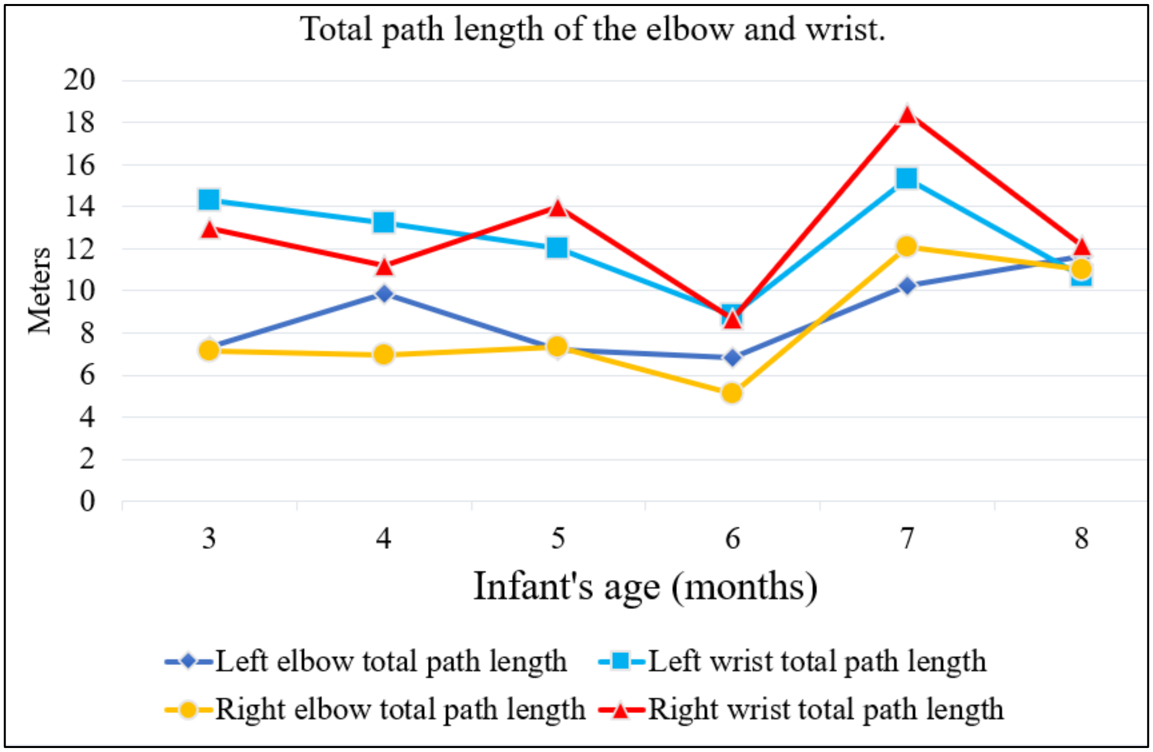


Figure 4. Calculated the distance between every two consecutive points. The sum of these points demonstrates the total path length of the elbow and wrist.

# Supplement E

Excerpt from: Kuo, H.-H. (2023). *Home assessment of grasp development in infants for fine motor delay* [Dissertation]. The Catholic University of America.

## References


1. Balta D, Kuo H, Wang J, et al. Characterization of Infants' General Movements Using a Commercial RGB-Depth Sensor and a Deep Neural Network Tracking Processing Tool: An Exploratory Study. *Sensors (Basel)*. 2022;22(19):7426. doi:10.3390/s22197426
2. MATLAB. 2020 [cited 2020 December 29, 2020]; R2020a:[Available from: https://www.mathworks.com/products/new_products/release2020a.html.
3. *West, B.T., Analyzing longitudinal data with the linear mixed model's procedure in SPSS. Evaluation & the Health Professions, 2009. 32(3): p. 207-228.*
